# ENPP1-Fc prevents neointima formation in generalized arterial calcification of infancy through the generation of AMP

**DOI:** 10.1038/s12276-018-0163-5

**Published:** 2018-10-29

**Authors:** Yvonne Nitschke, Yan Yan, Insa Buers, Kristina Kintziger, Kim Askew, Frank Rutsch

**Affiliations:** 1 Department of General Pediatrics, Münster University Children’s Hospital, Albert-Schweitzer-Campus 1, D-48149 Münster, Germany; 2 Cells in Motion Cluster of Excellence, Münster University, Münster, Germany; 3 Alexion Pharmaceuticals, 100 College St, New Haven, CT USA

## Abstract

Generalized arterial calcification of infancy (GACI) is associated with widespread arterial calcification and stenoses and is caused by mutations in *ENPP1*. *ENPP1* encodes for ectonucleotide pyrophosphatase/phosphodiesterase 1 (ENPP1), which cleaves ATP to generate inorganic pyrophosphate (PP_i_) and adenosine monophosphate (AMP) extracellularly. The current study was designed to define the prevalence of arterial stenoses in GACI individuals and to identify the mechanism through which ENPP1 deficiency causes intimal proliferation. Furthermore, we aimed to effectively prevent and treat neointima formation in an animal model of GACI through the systemic administration of recombinant human (rh)ENPP1-Fc protein. Based on a literature review, we report that arterial stenoses are present in at least 72.4% of GACI cases. We evaluated the effect of rhENPP1-Fc on *ENPP1*-silenced human vascular smooth muscle cells (VSMCs) and on induced intimal proliferation in Enpp1-deficient *ttw/ttw* mice treated with carotid ligation. We demonstrate that silencing *ENPP1* in VSMCs resulted in a tenfold increase in proliferation relative to that of cells transfected with negative control siRNA. The addition of rhENPP1-Fc, AMP or adenosine restored the silenced ENPP1-associated proliferation. In contrast, neither PP_i_ nor etidronate, a current off-label treatment for GACI, had an effect on VSMC proliferation. Furthermore, subcutaneous rhENPP1-Fc protein replacement was effective in preventing and treating intimal hyperplasia induced by carotid ligation in an animal model of GACI. We conclude that ENPP1 inhibits neointima formation by generating  AMP. RhENPP1-Fc may serve as an approach for the effective prevention and treatment of arterial stenoses in GACI.

## Arterial disease: promising protein replacement therapy in inherited disorder

A protein replacement therapy may prove useful in tackling calcification and narrowing of the arteries in babies with a severe genetic disorder. Generalized Arterial Calcification of Infancy (GACI) is a rare condition in which infants’ arteries become calcified and their blood vessels internally scarred. It often leads to congestive heart failure. The *ENPP1* gene encodes a protein that is crucial to preventing excess calcium build-up in the body. Mutations in the *ENPP1* gene lead to GACI, but no therapies for the condition exist. Now, Frank Rutsch at Muenster University Children’s Hospital in Germany and co-workers have shown that administering a protein replacement can inhibit blood vessel scarring and arterial clogging in GACI mice models and in human stem cell cultures. The protein replacement boosts production of a key metabolic molecule called adenosine monophosphate.

## Introduction

Generalized arterial calcification of infancy (GACI, MIM #208000) is a rare autosomal recessive disorder, and the disease frequency is one in 391,000^1^. The primary characteristics of GACI include severe calcification of the media of large and medium-sized arteries, accompanied by intimal proliferation leading to arterial stenoses within the first month of life^2^. GACI patients develop hypertension and myocardial ischemia, as well as severe congestive cardiac failure. Most affected patients die within the first half year of life^3–5^. Plasma levels of inorganic pyrophosphate (PP_i_) and ectonucleotide pyrophosphatase/phosphodiesterase 1 (ENPP1) enzymatic activity are extensively reduced in GACI patients^2^. Inactivating mutations in *ENPP1* (MIM *173335), have been identified as the underlying defect in approximately 75% of GACI cases^6,7^. ENPP1, a type II transmembrane glycoprotein, forms disulfide-bonded homodimers in the plasma membrane and in mineral-depositing matrix vesicles of osteoblasts and chondrocytes^8–10^. ENPP1 converts extracellular ATP to AMP, generating PP_i_. PP_i_ is a physiologic inhibitor of hydroxyapatite formation. PP_i_ regulates chondrogenesis and collagen I expression and synthesis and is therefore important for the prevention of soft tissue calcification^11–13^. The resulting AMP is hydrolyzed by the ecto-5-prime nucleotidase (CD73 or NT5E, MIM*129190) to adenosine and P_i_^14,15^.

Early generation bisphosphonates, which are synthetic analogues of PP_i_, have been used successfully to reduce the calcifications in GACI patients^6,16^. However, early death in infancy can occur even with bisphosphonate treatment^17^. Additionally, prolonged etidronate therapy has been related to osteonecrosis and osteomalacia in GACI patients^18^. Remarkably, the spontaneous resolution of arterial calcifications can be observed as the natural course of the disease in some GACI patients, even without bisphosphonate therapy^19^. Furthermore, no reduction of intimal hyperplasia has been reported. Orally administered PP_i_ has been shown to prevent soft tissue calcification in mouse models of GACI, but has not been shown to stop or reverse calcification that is already in progress^20^. The effect of orally administered PP_i_ on intima proliferation has not been investigated. Recently, enzyme replacement studies have shown to be effective for the prevention of arterial calcification in a mouse model of GACI, using recombinant ENPP1-Fc fusion protein^21^. The subcutaneous administration of ENPP1-Fc prevented mortality and soft tissue calcifications and improved the outcomes of the disease in Enpp1^asj/asj^ mice, an animal model of GACI.

Tiptoe-walking (*ttw/ttw*) mice develop periarticular and arterial calcifications, as well as progressive ectopic ossification of the spinal ligaments, in early life, due to a naturally occurring nonsense truncation mutation in *Enpp1*^22,23^. *Enpp1* knockout mice display an almost identical phenotype to that of *ttw/ttw* mice, with reduced levels of extracellular PP_i_, resulting in severe calcification of the cartilage and soft tissues, such as arterial walls^13,24^. Neither of the mouse models, *ttw/ttw* and *Enpp1*^−*/*−^, show myointimal proliferation per se. However, *Enpp1*^−*/*−^ mice show marked intimal vascular smooth muscle cell (VSMC) proliferation in response to arterial injury in vivo^25^. Thus far, it is not known whether injury-triggered intimal proliferation is also increased in *ttw/ttw* mice. In our study, we examined the prevalence of arterial stenoses in GACI cases, based on a literature survey, and we investigated different treatment options for inhibiting VSMC proliferation during ENPP1 deficiency. We demonstrated that the rhENPP1-Fc protein replacement is effective for inhibiting proliferation associated with the loss of ENPP1 in human induced pluripotent stem cell (hiPSC)-derived VSMCs and for preventing intimal hyperplasia in an animal model of GACI.

## Materials and methods

### Literature survey of published case reports

To evaluate the prevalence of myointimal proliferation and stenosis in patients with GACI, we surveyed available published case reports. Only case reports with detailed descriptions were included. Criteria for the identification of intimal proliferation and stenosis in case reports were as follows: histologic indication, imaging, such as angiography and Doppler, renovascular hypertension, and description of the arterial lumen using the terms narrowed, occluded, obstructed, or coarctation.

### Human material

For this study, we used plasma material from our international GACI registry^6^. Clinical and mutational data on the patients have been published previously^6^. The investigated patient plasma was derived from patients with disease causing mutations in *ENPP1*. The study protocol was approved by the Münster University Hospital Ethical Committee and conforms with the principles outlined in the Declaration of Helsinki. The parents of all subjects involved in this study gave informed written consent.

### Mice

The *ttw/ttw* mice used in this study have been described previously^22,23^. *ttw/ttw* mice were bred onto a C57BL/6J background for more than ten generations, and *ttw/ttw* and wild-type (WT) littermate control (male and female) animals were generated through heterozygous mating. The study was approved by the local committee for animal studies (Reg. Nos. 8.87-50.10.36.08 and 84-02.04.2015.A312) and was performed according to the guidelines from Directive 2010/63/EU of the European Parliament on the protection of animals used for scientific purposes.

### Plasma collection

Whole blood from GACI patients and healthy controls, as well as from *ttw/ttw* and WT mice (by cardiac puncture), was collected in syringes containing trisodium ethylenediaminetetraacetic acid (EDTA) and maintained on ice until the separation of plasma and erythrocytes by centrifugation (1000×*g*, 4 °C, 20 min) was performed. The plasma was then depleted of platelets by filtration (2200×*g*, 4 °C, 20 min) through a Centrisart I 300,000-kDa mass cutoff filter (Sartorius, Göttingen, Germany) and stored at −20 °C until further processing.

### PP_i_ and ATP determination

PP_i_ and ATP in human and mouse plasma was defined as described previously by Jansen et al.^26^. The PP_i_ level in the culture supernatant was measured using a radioactive assay. Master mix containing 49.6 mM Trizma acetate, 4.5 mM magnesium acetate tetrahydrate, 3.5 μM NAPD-Na_2_, 16.2 μM d-glucose-1,6-diphosphate, 6.6 μM uridine-5-diphosphoglucose, 0.002 U/μl phosphoglucomutase, and 0.003 U/μl glucose-6-phosphate dehydrogenase was first prepared and then 0.00118 U/μl Uridine 5′-diphosphoglucose pyrophosphorylase and 0.0002 µCi/μl Uridine diphospho-d-[6-3H] glucose was added. Then, 25 µl of sample was immediately added into 115 µl master mix and incubated for 30 min at 37 °C, after which 200 μl of cold 3% activated charcoal was added, followed by incubation for an additional 30 min at 4 °C. The radioactivity was measured using micro beta counter (Perkin Elmer, Hopkinton, MA, USA).

### Transfection of siRNA

hiPSC-derived VSMCs were purchased from ImStem Biotechology (Farmington, CT, USA). hiPSC-derived VSMCs were chosen to ensure an adequate amount of the same cells for all experiments. Primary rat VSMCs were prepared by using the enzymatic digestion of thoracic arteries from 3-week-old Sprague–Dawley rats. Transfection reagents were obtained from ThermoFisher Scientific (Waltham, MA, USA), except for siRNA targeting rat *ENPP1*, which was obtained from Sigma-Aldrich (St. Louis, MO, USA). Cells were seeded in a collagen type I-coated 60 mm dish or a regular polystyrene dish at a density of 3500 cells/0.32 cm^2^ in Smooth Muscle Growth Medium-2 (Lonza, Allendale, NJ, USA) or Vascular Cell Basal Medium (ATCC, Manassas, VA, USA), which is contained in the Vascular Smooth Muscle Cell Growth Kit (ATCC). After overnight culture, either siRNA targeting human *ENPP1* s10264 (Cat 4390824) and control siRNA (Cat 4390846) or siRNA targeting rat *ENPP1* (SASI Rn01_00111206) and control siRNA (Cat 4390847) were transfected into hiPSC-derived VSMCs or rat VSMCs, respectively, using Lipofectamine RNAiMAX overnight, according to the manufacturer’s instructions.

### Immunofluorescence

hiPSC-derived VSMCs were plated in the wells of 24-well plate at a density of 1.5 × 10e4 cells/well and were cultured overnight. Cells were fixed with 4% paraformaldehyde for 15 min, permeabilized with 0.5% Trition-X100 for 5 min, blocked with 5% goat serum for 1 h, and then stained using rabbit anti-calponin antibody (#ab46794, Abcam, Cambridge, USA) and mouse anti-smooth muscle myosin heavy chain 11 antibody (#ab683, Abcam, Cambridge, USA) at a 1:200 dilution for 1 h at 37 °C. The samples were then washed with PBS three times, followed by fluorescently labeled secondary antibodies, cyanine labeled goat anti-rabbit IgG (H + L) and Alexa Fluor 488-labeled goat anti-mouse IgG (H + L) (#ab6939 and #ab150113, respectively, Abcam, Cambridge, USA) at a 1:100 dilution for 1 h at room temperature. Paraffin embedded slices from the aorta of a GACI patient were deparaffinized, incubated with 0.1% trypsin for 15 min, 37 °C, and permeabilized with 0.1% Trition-X100 for 15 min. After a 1 h block in 2% BSA/ 5% donkey serum, samples were stained with a mouse anti-SMC specific α-actin antibody (HHF35, M0635, Dako, Jena, Germany) at a 1:100 dilution, overnight, followed by a Cy3 labeled donkey anti-mouse IgG (H + L) (#AP192C, Sigma-Aldrich, St. Louis, MO, USA) at a 1:100 dilution for 1 h at room temperature. Nuclei were stained for 5 min with DAPI. Images were obtained using an ApoTome fluorescent microscope (Zeiss, Oberkochen, Germany).

### Quantitative reverse transcription polymerase chain reaction (qRT-PCR)

Total RNA was extracted from hiPSC-derived VSMCs using the RNeasy Mini kit and QIAshredder (Qiagen Inc., Valencia, VA, USA). The isolated RNA was quantified using a Nanodrop2000 (ThermoFisher Scientific, Waltham, MA, USA) and reverse transcribed into cDNA using the High-Capacity cDNA Reverse Transcription Kit (ThermoFisher Scientific, Waltham, MA, USA). The resulting cDNA was amplified using the TaqMan Universal PCR Master Mix (Applied Biosystems, Foster City, CA, USA) and detected by real-time PCR using a QuantStudio^™^ 7 Flex System. TaqMan probes for human *ENPP1* (Hs01054038_m1) and a housekeeping gene, glyceraldehyde-3-phosphate dehydrogenase (*GAPDH*, Hs99999905_m1) were obtained from ThermoFisher Scientific (Waltham, MA, USA). The target gene expression level was normalized by the *GAPDH* level in each sample.

### Proliferation assay

Proliferation was quantified by [^3^H]-thymidine incorporation. Briefly, VSMCs transfected overnight with *ENPP1* siRNA or control siRNA were re-seeded at 2500 cells/well in a 96-well plate precoated with collagen type I. Cells were cultured in complete medium either with or without 300 µM ATP, in addition to the absence or presence of rhENPP1-Fc protein (0.01–5 µg/ml) (Alexion, New Haven, CT, USA), etidronate (0.1–100 µM), PP_i_ (1–300 µM), AMP (0.3–300 µM) or adenosine (0.3–300 µM) for 3 days. Culture medium was replaced daily. [^3^H]-thymidine was added during the last 18 h of culture.

### Carotid artery ligation

Left carotid artery ligation surgery was performed in 7–8 week-old WT (*n* = 7) and *ttw/ttw* mice (*n* = 21). Mice were anesthetized by isoflurane inhalation (Forene^®^, Abott GmbH & Co. KG, Wiesbaden), at an initial concentration of 1 l/min oxygen to 3 vol% isoflurane, maintaining a concentration of 0.6 l/min oxygen to 1–1.5 vol% isoflurane. Carprofen was used for analgesia (5 mg/kg bodyweight through a subcutaneous injection; Rimadyl^®^, Pfizer, Berlin, Germany). Left carotid arteries were exposed through a small midline incision in the neck and ligated with a 5-0 nylon silk suture approximately 2 mm proximal from the carotid bifurcation. All animals recovered well from the procedure and showed no signs of stroke.

### Recombinant human ENPP1-Fc fusion protein administration

To determine the effect of rhENPP1-Fc (Alexion, New Haven, CT, USA) on intimal hyperplasia, 6–7-week-old homozygous *ttw/ttw* mice were treated with either vehicle (20 mM HEPES, pH 7.3, 140 mM NaCl) or rhENPP1-Fc at 10 mg/kg bodyweight by subcutaneous injection every other day. In a preventive approach, the mice were treated for 7 days prior to carotid ligation, and treatment continued for 14 days postsurgery. Mice were euthanized using CO_2_ inhalation 14 days after carotid ligation. To determine whether ENPP1 could have a therapeutic effect if administered after the carotid ligation, 6- to 7-week-old *ttw/ttw* mice were subjected to carotid ligation and allowed to recover. rhENPP1-Fc treatment (10 mg/kg bodyweight subcutaneously injected every other day) was initiated 7 days after carotid ligation and continued for 7 days until the carotid arteries were harvested at 14 days postligation. Carotid arteries were fixed with 4% paraformaldehyde in PBS for morphological analyses.

### Histological and morphological analyses of ligated carotid arteries

Serial sections (sections of 5 µm each) were collected. For morphometrical measurements of the ligated carotid arteries, sections immediately proximal of the ligation site were taken. By using every fifth section, a total of 12 sections per animal were analyzed proximal from the ligation site, spanning a distance of approximately 250 µm. Morphometric analyses were performed by using Elastica van Gieson stain (Roth, Karlsruhe, Germany). ImageJ software was used to measure the circumference of the external elastic lamina, the internal elastic lamina and the luminal border. The medial area, the intimal area and the intima/media ratio (*I*/*M* ratio) were calculated.

### Statistical analyses

Statistical analyses were performed using Student’s *t* test (unpaired two-sample testing for means). Comparisons of multiple groups used one-way ANOVA, followed by the Bonferroni’s post hoc test, performed with GraphPad Prism software version 7. Probability values of *p* < 0.05 were considered significant.

## Results

### Most GACI patients develop arterial stenoses

We evaluated 132 case reports that included 199 published GACI patients (Supplemental reference list). A diagnosis of GACI was confirmed if imaging studies showed calcification of the great or medium-sized arteries in infancy or if histology showed arterial calcification at the level of the *lamina* *elastica* *interna*. Of these individuals, 144 were described as having myointimal hyperplasia and/or arterial stenoses, representing 72.4% of evaluated cases. The remaining 55 GACI cases (27.6%) were not described as having intimal proliferation or arterial stenoses.

### **ENPP1 deficiency leads to decreased PP**_**i**_**and elevated ATP plasma levels**

**Fig. 1:**
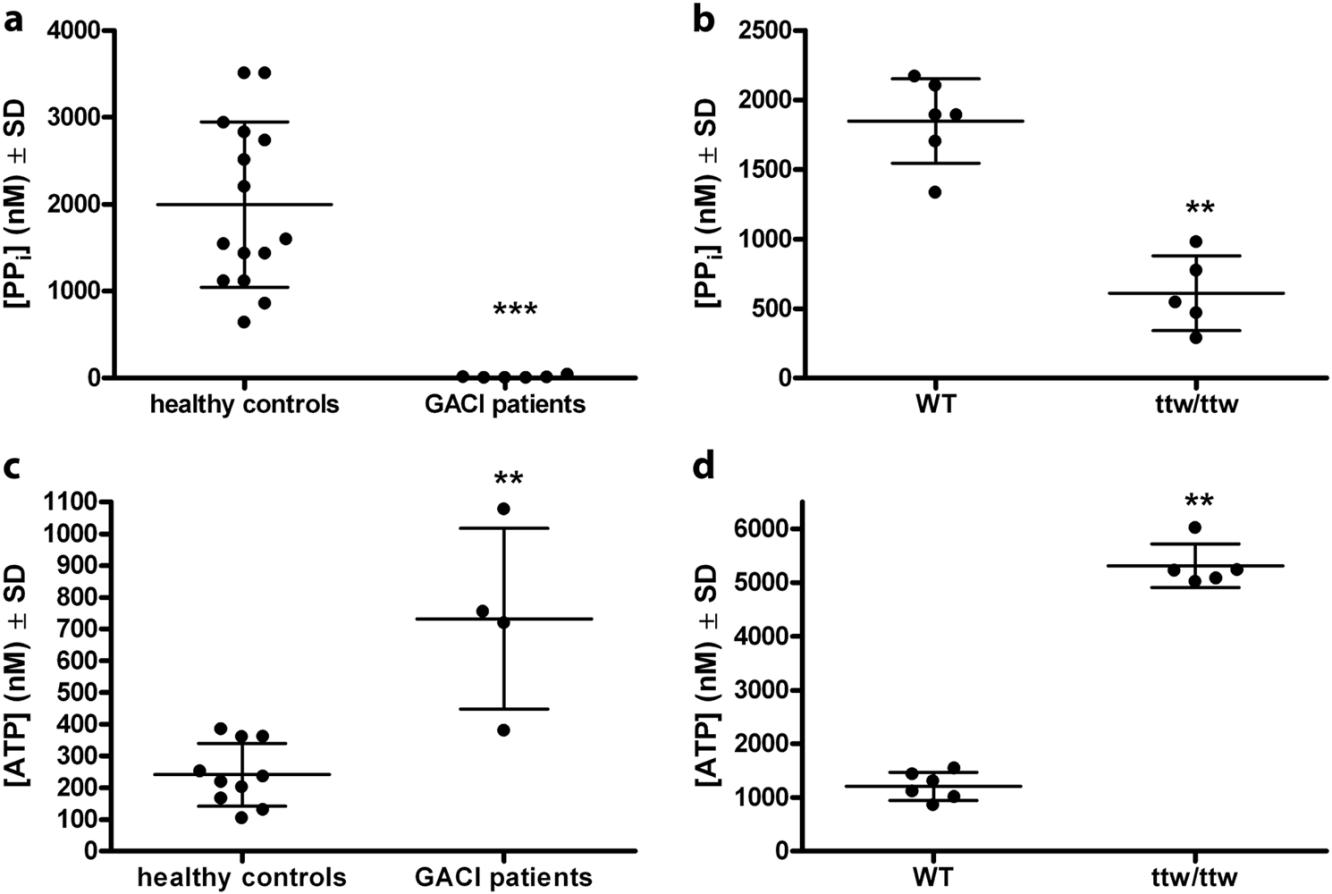
ENPP1 deficiency leads to decreased levels of PP_i_ and elevated levels of ATP in the plasma of GACI individuals and *ttw/ttw* mice. Plasma levels of PP_i_ (**a**, **b**) and ATP (**c**, **d**) were determined from healthy controls (*n* = 10) and GACI patients (*n* = 4) (**a**, **c**) and from 7-week-old WT (*n* = 6) and *ttw/ttw* mice (*n* = 5) (**b**, **d**). Plasma levels of ATP from healthy controls (**c**) correspond to normal human EDTA plasma concentrations from previous publications^27^. Values are presented as the mean ± SD. ^**^p < 0.005, ^***^p < 0.001 (Student’s *t* test, unpaired two-sample testing for means)

The plasma of GACI patients and *Enpp1*-deficient *ttw/ttw* mice was evaluated with regards to PP_i_ and ATP levels. We found that the PP_i_ plasma levels of GACI patients were dramatically reduced or non-detectable compared to healthy controls (Fig. 1a, *p* < 0.001). Similarly, PP_i_ levels in the plasma of *ttw/ttw* mice were significantly reduced compared to those of WT mice (Fig. 1b, *p* < 0.001). Interestingly, the plasma ATP levels of GACI patients and those of *ttw/ttw* mice were significantly increased compared to controls (Fig. 1c, d, threefold and fourfold, increases, *p* < 0.05 and *p* < 0.001, respectively). The plasma levels of ATP from healthy controls (Fig. 1c) corresponded with normal human EDTA plasma concentrations reported in previous publications^27^.

### Silencing of ENPP1 by siRNA increases the proliferation of hiPSC-derived VSMCs

**Fig. 2:**
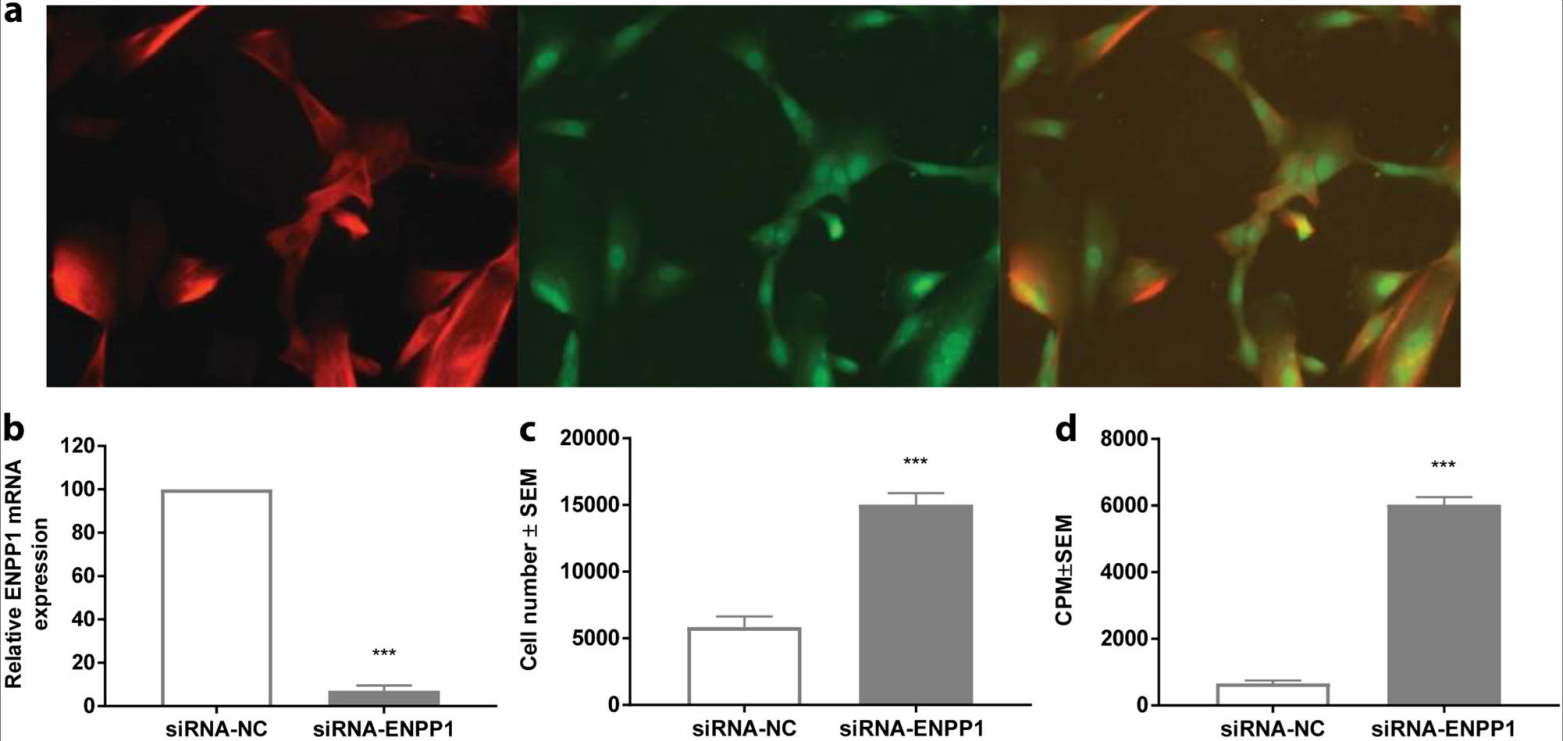
Silencing of *ENPP1* by siRNA in hiPSC-derived VSMCs results in a reduction of *ENPP1* RNA expression and an increase in hiPSC-derived VSMCs proliferation. SMC markers calponin (first panel) and SM-MHC11 (second panel) confirmed the SMC phenotype of hiPSC-derived VSMCs (**a**). hiPSC-derived VSMCs were transfected overnight with *ENPP1* siRNA or control siRNA, followed by 24-h starvation in 0.25% FBS. Then, the cells were cultured in basal media. *ENPP1* RNA expression (**b**) and hiPSC-derived VSMCs cell number (**c**), and proliferation (**d**) were investigated. **b**, **c** Values are presented as the mean ± SEM, *n* = 4; ^***^p < 0.001 vs. control nontargeting siRNA transfected cells. **d** Cell proliferation was evaluated by [^3^H] thymidine uptake. [^3^H]thymidine was added in the last 18 h of culture. The results are expressed as the CPM ± SEM, *n* = 4. ^***^p < 0.001 vs. control nontargeting siRNA transfected cells (Student’s *t* test, unpaired two-group testing for means)

VSMCs are the most prominent cell types found in areas of myointimal proliferation in GACI patients (Supplemental Fig. 1, upper panel). To explore whether ENPP1 is important for VSMC proliferation, we applied siRNA-induced knockdown of ENPP1 in hiPSC-derived VSMCs. hiPSC-derived VSMCs were shown to express the VSMC markers calponin and SM-MHC11 (Fig. 2a). The transient transfection of *ENPP1*-targeting siRNA resulted in an approximately 90% reduction of *ENPP1* mRNA-expression compared to nontargeting siRNA (Fig. 2b). The knockdown of ENPP1 increased the hiPSC-derived VSMC cell number by approximately threefold (Fig. 2c) and proliferation by approximately tenfold (Fig. 2d).

### rhENPP1-Fc but not bisphosphonates nor PP_i_ inhibits accelerated proliferation in *ENPP1*-silenced VSMCs

**Fig. 3:**
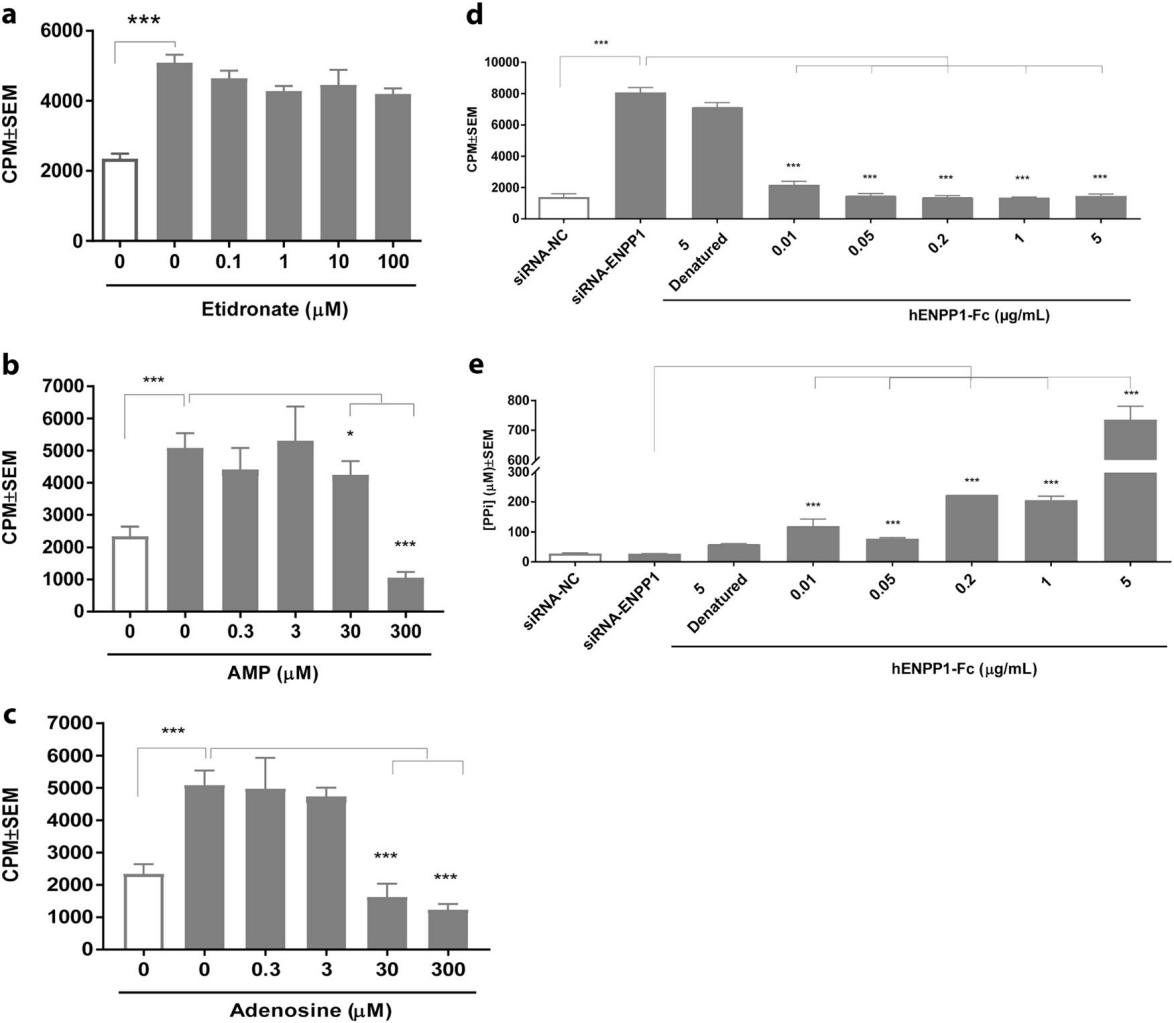
Effects of bisphosphonates (**a**), AMP (**b**), adenosine (**c**), and rhENPP1-Fc (**d**) on accelerated proliferation in *ENPP1*-silenced hiPSC-derived VSMCs. hiPSC-derived VSMCs were transfected overnight with *ENPP1* siRNA or control siRNA, followed by 24-h starvation in 0.25% FBS. Then, cells were cultured in basal media, supplemented with etidronate (0.1–100 µM, **a**), AMP (0.3–300 µM, **b**), and adenosine (0.3–300 µM, **c**) for 3 days before determining proliferation. Cell proliferation was evaluated by [^3^H] thymidine uptake. [^3^H]thymidine was added in the last 18 h of culture. Extracellular PP_i_ levels in the cell culture medium of cells supplemented with 300 µM ATP and rhENPP1-Fc (0.01–5 µg/ml, **d**) were determined (**e**). Values are presented as the mean ± SEM, *n* = 4. ^***^p < 0.001 (one-way ANOVA multiple group comparison followed by the Bonferroni’s post hoc test)

Transfected hiPSC-derived VSMCs were supplemented with etidronate, adenosine, AMP, or rhENPP1-Fc, and transfected rat VSMCs were supplemented with etidronate, PP_i_, adenosine, or AMP, for 3 days before determining proliferation. Etidronate was not able to inhibit ENPP1-knockdown-induced proliferation in hiPSC-derived VSMCs or rat VSMCs (Fig. 3a and Supplemental Fig. 2a, respectively). Further, the addition of PP_i_ to *ENPP1*-silenced rat VSMCs could not inhibit proliferation (Supplemental Fig. 2b). Interestingly, AMP, the product of ENPP1-mediated ATP hydrolysis (and a side-product of PP_i_ generation), was able to inhibit *ENPP1*-knockdown-induced proliferation at a concentration of 300 µM (Fig. 3b, *p* < 0.001, and Supplemental Fig. 2c, *p* < 0.01, respectively). At this concentration, AMP reduces proliferation by approximately 70%, to a level lower than that of nonsilenced hiPSC-derived VSMCs (Fig. 3b). In addition, adenosine, which is generated downstream of ENPP1-mediated ATP hydrolysis, is capable to inhibit the proliferation of *ENPP1*-silenced hiPSC-derived VSMCs by approximately 70% at a concentration of 30 µM (Fig. 3c, *p* < 0.001). In rat VSMCs, adenosine reduced the *ENPP1*-knockdown-induced proliferation by approximately 50% at a concentration of 300 µM (Supplemental Fig. 2d, *p* < 0.01). The addition of rhENPP1-Fc inhibited *ENPP1*-knockdown-induced proliferation by 75%, comparable to the level of silenced hiPSC-derived VSMCs (Fig. 3d, *p* < 0.001). The inhibition of proliferation was achieved at a concentration of only 0.01 µg/ml. The extracellular PP_i_ levels in cell culture media were significantly increased following the addition of rhENPP1-Fc (Fig. 3e, *p* < 0.001).

### ENPP1 deficiency increases neointimal lesion formation after carotid ligation injury in *ttw/ttw* mice

Representative stained sections from either 100 or 200 µm caudal from the ligation in *ttw/ttw*-mice and genetically matched control WT mice showed that, in WT mice, the carotid ligation caused intimal hyperplasia, resulting in the narrowing of the lumen, with more severe narrowing closer to the ligature (100 µm) and less severe occlusion further away (200 µm) (Supplemental Fig. 3a). In contrast, in *ttw/ttw* mice the degree of intimal hyperplasia appeared to be increased, as the lumen at 200 µm caudal from the ligation was almost completely occluded. The neointimal area primarily consisted of VSMCs (Supplemental Fig. 1, lower panel). Quantitative analyses of sequential sections of ligated common carotid arteries showed that *ttw/ttw* mice had significantly increased neointimal proliferation compared to WT mice after ligation-induced vascular remodeling for 14 days (Supplemental Fig. 3b, *p* < 0.001), but not thickened medial areas (Supplemental Fig. 3c). Correspondingly, the *I*/*M* ratio of *ttw/ttw* mice was markedly increased compared with WT mice (Supplemental Fig. 3d, *p* < 0.001). Right nonligated carotids from all mice had no measurable neointima.

### rhENPP1-Fc administration prevents and effectively treats neointima formation following arterial injury

**Fig. 4:**
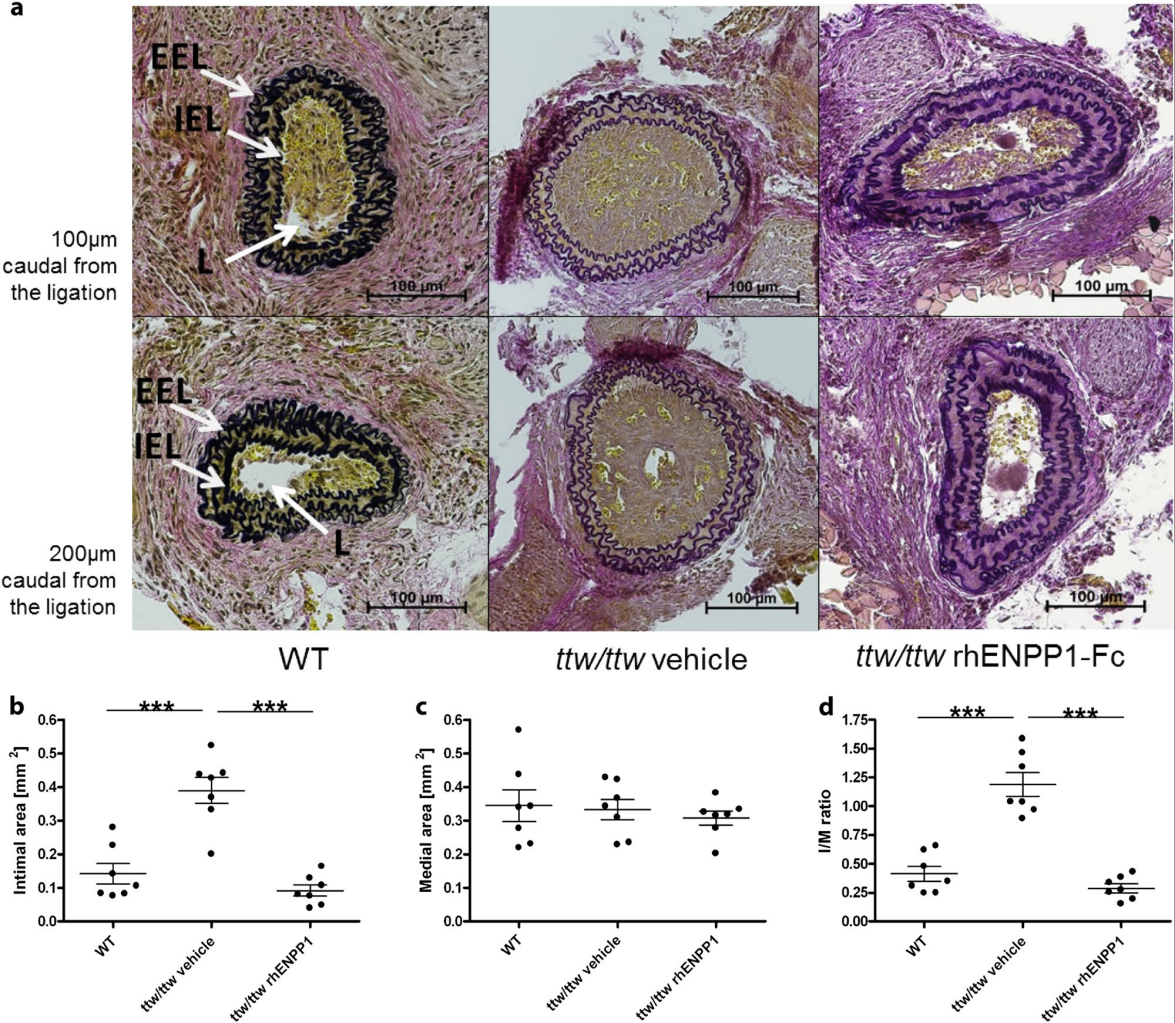
Administration of rhENPP1-Fc prevents intimal proliferation after carotid ligation in *ttw/ttw* mice. RhENPP1-Fc treatment was started 7 days prior to carotid ligation, and serial sections of the left carotid arteries were taken 14 days after carotid ligation. Histological analysis (Von Gieson’s stain) was performed on sections taken either 100 (upper panel) or 200 (lower panel) µm from the point of ligation from WT, vehicle-treated *ttw/ttw* or rhENPP1-treated *ttw/ttw*- mice, shown from left to right (**a**). The internal elastic lamina (IEL), external elastic lamina (EEL), and lumen (L) are indicated by arrows. The scale bar represents 100 µm. Morphometric quantitation was performed on intimal (**b**) and medial (**c**) areas, and the *I*/*M* ratio was calculated (**d**). Values are presented as the mean ± SEM, *n* = 7 each group, ^***^p < 0.001 (one-way ANOVA multiple group comparison followed by the Bonferroni’s post hoc test)

**Fig. 5:**
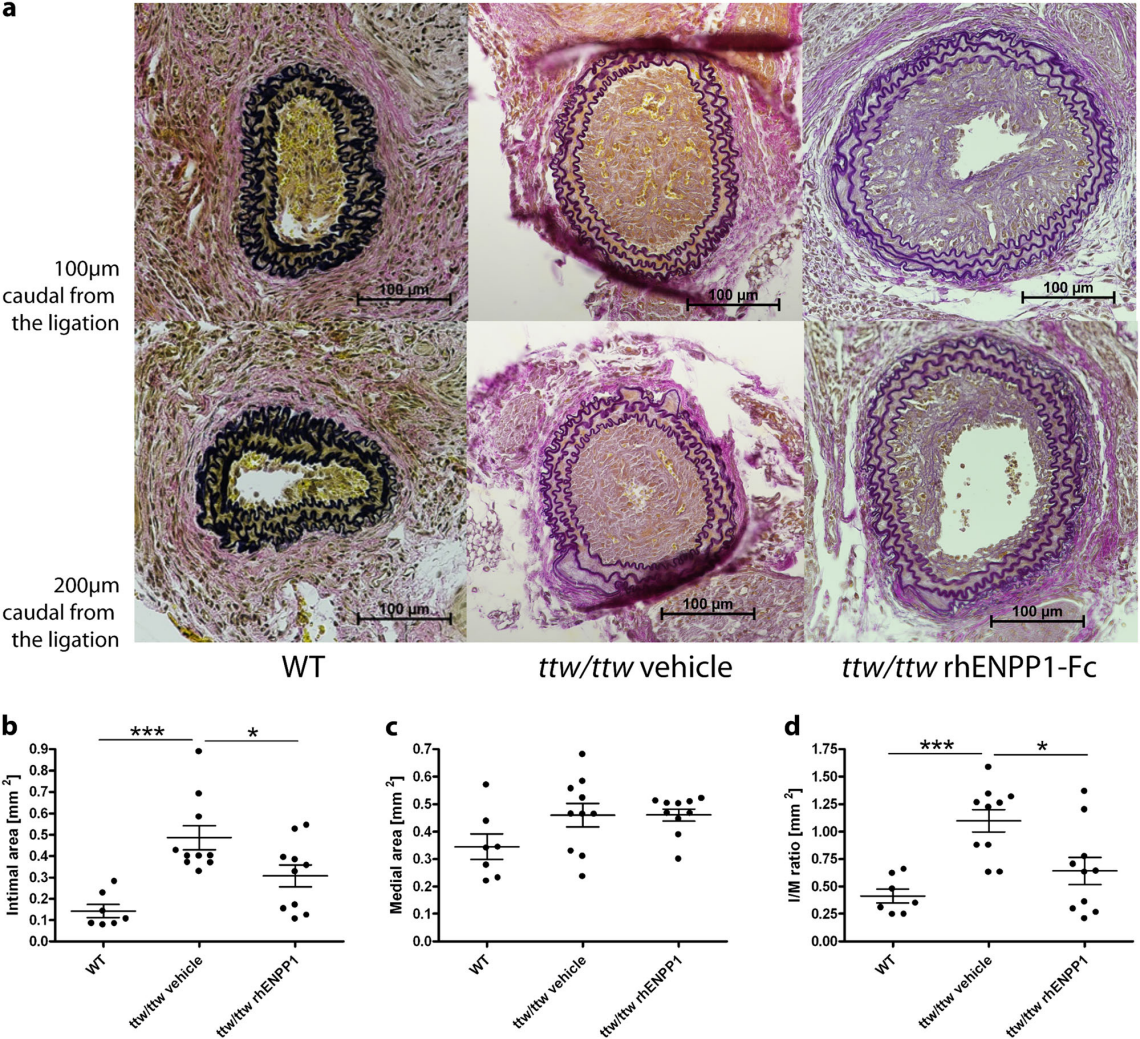
Therapeutic administration of rhENPP1-Fc inhibits intimal proliferation after carotid ligation in *ttw/ttw* mice RhENPP1-Fc treatment was started 7 days after carotid ligation, and serial sections of the left carotid arteries were taken 14 days after carotid ligation. Histological analysis (Von Gieson’s stain) was performed on sections taken either 100 (upper panel) or 200 (lower panel) µm from the point of ligation from WT, vehicle-treated *ttw/ttw* or rhENPP1-treated *ttw/ttw*− mice, shown from left to right (**a**). The scale bar represents 100 µm. Morphometric quantitation was performed on intimal (**b**) and medial (**c**) areas, and the *I*/*M* ratio was calculated (**d**). Values are presented as the mean ± SEM, *n* = 7 for WT, *n* = 10 for vehicle-treated *ttw/ttw* or rhENPP1-treated *ttw/ttw*- mice, ^***^p < 0.001, ^*^p < 0.05 (one-way ANOVA multiple group comparison followed by the Bonferroni’s post hoc test)

To determine the preventive effect of rhENPP1-Fc on the accelerated intimal hyperplasia in *ttw/ttw* mice, the mice were treated with either vehicle or rhENPP1-Fc for 7 days prior to carotid ligation, and treatment continued for 14 days postsurgery. The *ttw/ttw* mice treated with rhENPP1-Fc showed greatly reduced intimal hyperplasia than those treated with vehicle, approaching the degree observed in WT animals (Fig. 4a). The results of quantitative analyses of the neointimal and medial areas, as well as the *I*/*M* ratio of ligated common carotid arteries obtained in vehicle-treated *ttw/ttw* mice were similar to those of *ttw/ttw* mice without treatment. The intimal area of *ttw/ttw* mice receiving subcutaneous rhENPP1-Fc was significantly reduced compared to vehicle-treated *ttw/ttw* mice (Fig. 4b, *p* < 0.001), whereas the medial area, between the external and internal lamina, remained constant (Fig. 4c). The *I*/*M* ratio showed a statistically significant decrease in rhENPP1-Fc treated *ttw/ttw* mice compared to vehicle-treated *ttw/ttw* mice (Fig. 4d, *p* < 0.001), approaching the degree observed in WT animals. An assessment of the therapeutic effects of rhENPP1-Fc was initiated starting 7 days postligation, when myointimal proliferation was definitely present. Therapeutic treatment with rhENPP1-Fc beginning at this point showed benefits, as the degree of luminal occlusion at both 100 and 200 µm was less than that of vehicle-treated animals 14 days postligation (Fig. 5a). The quantitative analyses revealed that rhENPP1-Fc treated *ttw/ttw* mice had a significantly smaller intimal area compared to vehicle-treated *ttw/ttw* mice (Fig. 5b, *p* < 0.05), whereas no significant difference was observed between rhENPP1-Fc treated *ttw/ttw* mice and nontreated WT mice. The medial area, between the external and internal lamina, remained constant between WT, vehicle-treated and rhENPP1-Fc treated *ttw/ttw* mice (Fig. 5c). The I/M ratio of vehicle-treated *ttw/ttw* mice was increased compared to the levels of WT and rhENPP1-Fc treated *ttw/ttw* mice (Fig. 5d, *p* < 0.001 and *p* < 0.05, respectively). The *I*/*M* ratio was not significantly different between WT and rhENPP1-Fc treated *ttw/ttw* mice.

## Discussion

Although the pathophysiologic role of ENPP1-mediated PP_i_ generation and the impairment of extracellular purinergic metabolism in GACI has come to light, there is still no specific therapeutic option for treating GACI. Especially in recent years, different approaches for treating GACI have been intensively investigated, including early generation bisphosphonates^6,28,29^, orally administered PP_i_^20^ and soluble recombinant ENPP1-Fc protein^21^. However, all of these studies focused on treating arterial calcification, while the most prominent complications for GACI patients are severe congestive cardiac failure, hypertension and myocardial ischemia due to myointimal proliferation and arterial stenosis^1,6^. In our literature survey, approximately 72% of published GACI cases display intimal hyperplasia and/or arterial stenoses. However, the percentage of patients presenting with vascular occlusion may be even higher, as not all of the cases studied had been investigated for arterial stenoses. Therefore, vascular occlusion significantly contributes to morbidity and mortality. Here, we demonstrate for the first time that the enzyme replacement of ENPP1 inhibits accelerated VSMC proliferation and arterial stenoses caused by ENPP1 deficiency.

Dysregulated VSMC function is fundamental to myointimal hyperplasia: the proliferation of medial SMCs, migration to the intima, and the proliferation of intimal cells are sequential steps in the formation of myointimal hyperplasia^30^. We showed that the knockdown of *ENPP1* in hiPSC-derived VSMCs leads to significant increases in cell numbers and proliferation. Interestingly, ENPP1-deficient mouse models, including *ttw/ttw* mice, do not develop myointimal proliferation per se. However, in our study, injury-triggered myointimal proliferation was significantly increased in ENPP1-deficient *ttw/ttw* mice when compared to WT mice within 14 days after carotid artery ligation. Serrano et al.^25^ demonstrated that the increased proliferation observed during ENPP1 deficiency is associated with dysregulated VSMC function and ER stress. The authors assumed that there were biologic effects of ENPP1 deficiency on VSMCs other than osteochondral differentiation and calcification. Arterial calcifications in ENPP1 deficiency are clearly due to decreased extracellular PP_i_ concentrations^2,21,31,32^. It has been shown that orally administered PP_i_ can fully inhibit arterial calcification in  ENPP1-deficient mice^20^. Interestingly, *ENPP1* knockdown related accelerated proliferation in VSMCs could not be inhibited by the addition of PP_i_ or bisphosphonates, i.e., the synthetic analogues of PP_i_, indicating a mechanism that is independent of ENPP1-mediated extracellular PP_i_ concentration. This result is in accordance with clinical studies in GACI individuals showing that treatment with bisphosphonates increases the rate of survival, but has no influence on intimal proliferation or vascular stenosis in ENPP1-deficient patients^1,6,33^. Recently, it has been shown that the treatment of Enpp1^asj/asj^ mice with recombinant ENPP1 elevated extracellular PP_i_ levels and inhibited arterial calcification^21^. However, myointimal proliferation was not investigated in that study.

We demonstrated that supplementation with the recombinant human enzyme ENPP1 fully inhibited the accelerated proliferation of *ENPP1*-silenced hiPSC-derived VSMCs. Furthermore, in our study, the ENPP1 enzyme replacement prevented and effectively treated myointimal proliferation and stenosis in carotid ligated *ttw/ttw* mice, comparable to the proliferation level of ligated WT mice. This result demonstrates that the presence of ENPP1 prior to and after carotid ligation protects against intimal hyperplasia. Additionally, we were able to show, that rhENPP1-Fc has the potency to reverse intimal proliferation that is already present.

ENPP1 plays a role in purinergic metabolism and signaling by removing ATP and synthesizing AMP, which is further hydrolyzed to extracellular adenosine by CD73^14,15,34^. It is known that elevated extracellular ATP levels lead to accelerated proliferation^35,36^. In fact, ENPP1-deficient GACI patients, as well as *ttw/ttw* mice, demonstrated elevated plasma ATP levels compared to healthy controls and WT mice, respectively. Presumably, elevated extracellular ATP levels due to ENPP1 deficiency could trigger increased VSMC proliferation, leading to intimal stenosis. Furthermore, the proliferation induced by *ENPP1* knockdown in hiPSC-derived VSMCs was inhibited by AMP, as well as its product adenosine, in a dose-dependent manner, and adenosine inhibited proliferation at a lower dose than AMP. The downstream ATP hydrolysis product AMP is known to inhibit VSMC proliferation through the AMP-activated protein kinase pathway. The effect of AMPK on VSMC proliferation might be mediated by two pathways. Phosphorylated AMPK inhibits the mTOR complex 1 pathway, leading to cell cycle arrest^37^. Furthermore, AMPK mediates proliferation inhibition through the induction of the cell cycle inhibitors p53 and p21^CIP^^38^. These events inhibit cell proliferation and protein synthesis. Correspondingly, adenosine has been shown to inhibit VSMC proliferation induced by different substances, such as mitogen or FCS, in vitro and injury-induced neointima formation in vivo^39–43^. Adenosine interacts with G protein-coupled P1A_2_ receptors, with effects that are opposite those triggered by ATP. Most likely, the binding of adenosine to P1A_2_ receptors activates adenylyl cyclase, which results in a significant increase in cAMP levels and the stimulation of protein kinase A, which stimulates multiple signaling pathways, leading to the inhibition of proliferation^39,44^. However, the underlying mechanism(s) in GACI are unknown. It is not known whether excess ATP or insufficient AMP/adenosine levels induce proliferation in GACI, and the participation of other mechanisms cannot be ruled out.

ENPP1 is the major enzyme that is responsible for extracellular ATP hydrolysis and the generation of AMP^45^, the precursor of adenosine^34^. Apparently, ENPP1 is not only essential for the generation of extracellular PP_i_ but also for the cleavage of extracellular ATP for the creation of AMP and downstream hydrolysis products, such as adenosine. Interestingly, CALJA patients (calcification in joints and arteries, MIM#211800) with deficiencies in CD73 develop extensive vascular obstructions in the lower extremities in adulthood^14,15^. Additionally, CD73 knockout mice develop increased myointimal proliferations after wire-induced injuries^46^. The pathological findings of myointimal proliferation and stenosis are obviously more widespread and occur at an earlier age of onset in GACI patients than in CALJA patients. These differences might be because, in GACI patients, both proliferation inhibitors, adenosine and its precursor AMP, are deficient, whereas CALJA patients only show a reduction in extracellular adenosine levels due to deficient CD73 activity. In this respect, whether extracellular AMP levels are increased in CALJA patients must be investigated. Another player in extracellular ATP / PP_i_ metabolism that is involved in the regulation of VSMC proliferation is the ectonucleoside triphosphate diphosphohydrolase-1 (ENTPD1 or CD39, MIM*601752). CD39 hydrolyzes ATP to ADP and then to AMP by a two-step process^47^. The loss of CD39 activity leads to an accumulation of extracellular ATP^48^. CD39 deficient VSMCs show increased proliferation in vitro^49^. Interestingly, CD39 knockout resulted in decreased neointimal formation after carotid injury in vivo, likely due to the impaired migration response of CD39 deficient VSMCs^49^. However, the overexpression of CD39 reduced myointimal proliferation following angioplasties in rats^50^. The analogical, systemic administration of human soluble CD39 protected against neointimal thickening following vascular injury in mice^51^. The authors hypothesized the direct inhibition of nucleotide-induced recruitment and SMC proliferation caused by the normalizing of extracellular nucleotide levels following the administration of soluble CD39.

**Fig. 6:**
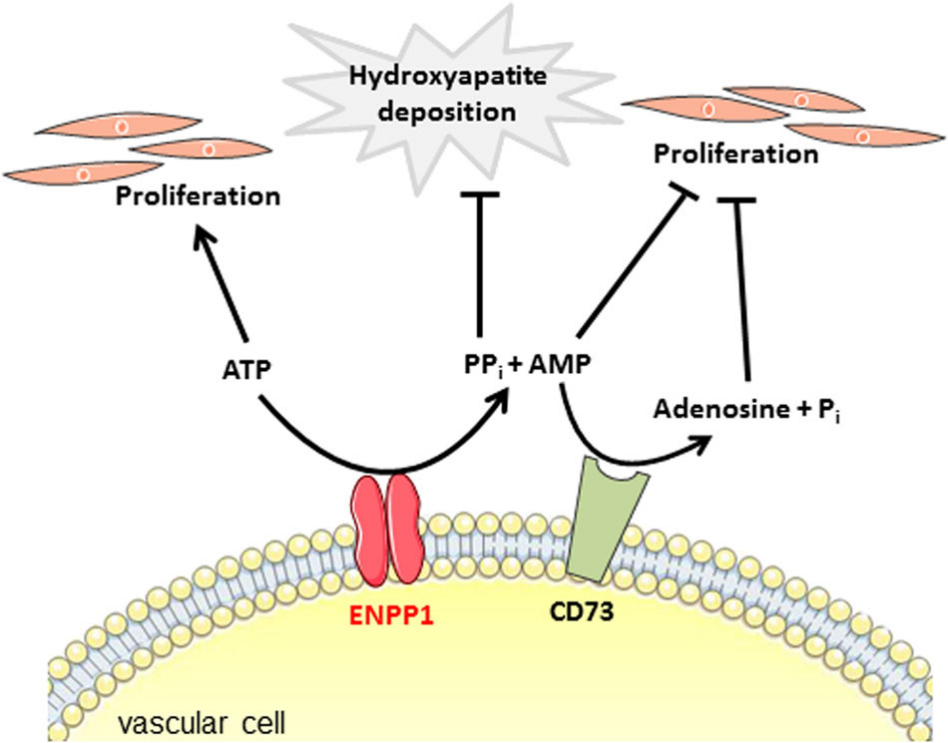
The two roles of ENPP1: inhibiting arterial calcification and myointimal proliferation by modulating extracellular PP_i_ and ATP metabolism. The transmembrane ectoenzyme ENPP1 converts extracellular ATP to AMP and thereby generates PP_i_. AMP is further hydrolyzed by CD73 (5-exonucleotidase) to adenosine and P_i_. PP_i_ is a physiologic inhibitor of hydroxyapatite formation, regulates chondrogenesis and is therefore important in the prevention of soft tissue calcification. A decreased PP_i_/P_i_ ratio leads to calcification. Elevated extracellular ATP levels lead to increased VSMC proliferation, while AMP and adenosine are known to inhibit VSMC proliferation. In ENPP1 deficiency, independent of low PP_i_ levels, proproliferating extracellular ATP levels increase, whereas antiproliferating AMP and adenosine levels decrease, leading to accelerated VSMC proliferation and arterial stenosis. We conclude that a decreased PP_i_/P_i_ ratio leads to ectopic calcification, whereas an increased extracellular ATP/AMP ratio leads to increased VSMC proliferation and arterial stenosis in ENPP1 deficiency

During ENPP1 deficiency and independent of low PP_i_ levels, proproliferating extracellular ATP levels increase, whereas antiproliferating AMP and adenosine levels decrease, leading to accelerated VSMC proliferation and stenosis. We conclude that an increased extracellular ATP/AMP ratio leads to increased VSMC proliferation and arterial stenosis during ENPP1 deficiency (Fig. 6). Through the administration of recombinant human ENPP1, both extracellular ATP and AMP levels might be normalized, leading to the antiproliferative signaling of AMP and adenosine and the inhibition of VSMC proliferation. We speculate that, in contrast to orally administered PP_i_ or bisphosphonates, which serve as calcification inhibitors in GACI related arterial calcification^6,20^, soluble rhENPP1-Fc not only acts on enhanced ectopic calcification by elevating extracellular PP_i_ levels but also represses VSMC proliferation by cleaving extracellular ATP. Interestingly, it appears that low concentrations of rhENPP1-Fc were maximally effective at reducing VSMC proliferation. However, low concentrations only caused a small increase in PP_i_ concentrations. Generated PP_i_ levels should reflect ENPP1 function and the generation of AMP. These data imply that low rhENPP1-Fc is sufficient to generate maximally effective concentrations of AMP and/or adenosine with only a small increase in PP_i_.

It is important to recognize that the extremes of our results may be due to experimental conditions. Mice do not develop myointimal proliferation *per se*. For our investigations, we had to use an artificial in vivo carotid ligation system, leading to injury-induced VSMC proliferation, which does not reflect the natural myointimal proliferation in GACI. Because WT mice were not treated with rhENPP1-Fc, off target effects cannot be excluded. Our experimental protocol was not directed to investigate long-term treatment with rhENPP1-Fc.

Our results suggest a novel role for ENPP1, namely decreasing the extracellular ATP/AMP ratio to inhibit VSMC proliferation, most likely by influencing purinergic signaling.

To this point, no current medical strategies for treating myointimal hyperplasia in GACI exist. Therapeutics used to treat GACI patients are directed to reduce arterial calcification and are typically not effective in inhibiting intimal proliferation, as has been shown using bisphosphonates^33^. In our study, we were able to show that increased VSMC proliferation due to ENPP1 deficiency can be inhibited by the administration of recombinant human ENPP1-Fc protein in vitro and in vivo. Our findings have implications for ENPP1 enzyme replacement as a potential therapeutic approach for treating intimal hyperplasia in GACI.

## Electronic supplementary material

Supplemental Material

Supplemental Figure 1

Supplemental Figure 2

Supplemental Figure 3
