# Supplementary material for: ENPP1-Fc prevents neointima formation in generalized arterial calcification of infancy through the generation of AMP
Source: Exp Mol Med. 2018 Oct 29;50(10):139. doi: 10.1038/s12276-018-0163-5 (PMC6204430; doi:10.1038/s12276-018-0163-5)
Supplement: Supplementary file 1 — Supplemental Material [file 12276_2018_163_MOESM1_ESM.pdf]

Supplementary Information

ENPP1-Fc prevents neointima formation in generalized arterial calcification of infancy through the generation of AMP

Yvonne Nitschke[1,3]*, Yan Yan[2]*, Insa Buers[1,3], Kristina Kintziger[1], Kim Askew[2], Frank Rutsch[1,3]#

*These authors contributed equally to this work.
#Corresponding Author

Affiliations:
[1]Department of General Pediatrics, Münster University Children's Hospital, Albert-Schweitzer-Campus 1, D-48149 Münster, Germany

[2]Alexion Pharmaceuticals, 100 College St, New Haven, USA

[3]Cells in Motion Cluster of Excellence, Münster University, Münster, Germany

Supplemental Figure legends

Supplemental Figure 1: VSMCs are the most prominent cells in areas of myointimal proliferation in GACI patients. Immunofluorescence staining of SMC specific α-actin (red) is demonstrated in the intima of the aorta of a GACI patient (upper panel) and ligated left carotid of *ttw/ttw* mouse (lower panel), displaying myointimal proliferation. Autofluorescence is shown in green and nuclei in blue. Dotted lines indicate the intimal:medial boundary.

Supplemental Figure 2: Effect of bisphosphonates (etidronate) (a), PP_i (b), AMP (c), and adenosine (d) on accelerated proliferation in *ENPP1* silenced primary rat VSMC. Primary rat VSMC were transfected overnight with *ENPP1* siRNA or control siRNA followed by 24 hour starvation in 0.25% FBS. Then cells were reseeded in wells of 96 well plate, cultured in basal media, supplemented with, etidronate (0.1 – 100µM, **a**), PP_i (1 – 300µM, **b**), AMP (100 – 300 µM, **c**), adenosine (100 – 300µM, **d**) for 3 days before determine proliferation. Cell proliferation was evaluated by [3H] thymidine uptake. [3H]-thymidine was added in the last 18 hours of culture. Results are expressed as CPM ± SEM, n=4. ***p<0.001, **p<0.005 (One way Anova, multiple group comparison).

Supplemental Figure 3: Accelerated intimal hyperplasia is present in *ttw/ttw*–mice after carotid ligation compared to WT-mice. Histological analysis (Von Gieson's stain) are made of sections either 100 (upper panel) or 200 (lower panel) µm from point of ligation from WT- and *ttw/ttw*- mice from left to right, respectively (**a**). The internal elastic lamina (IEL), external elastic lamina (EEL) and lumen (L) are indicated by arrows. The scale bar represents 100 µm. Morphometric quantitation was done of intimal (**b**) and medial (**c**) areas and the I/M ratio (**d**). Values are means ±SEM, n=7 each group, **p<0.005 (Student's *t*-test, un-paired two-group testing for means).

Supplementary Reference list of Case Reports on Generalized Arterial Calcification of Infancy

1. Agrawal G, Chintala K. Antenatal diagnosis of idiopathic arterial calcification with hydrops fetalis. *Eur Heart J Cardiovasc Imaging* 2015; **16**(7)**:** 816.

2. Althen B, Wiedersberg H. [Clinical aspects, morphology and heredity of arteriopathia calcificans infantum]. *Kinderarztl Prax* 1985; **53**(3)**:** 127-32.

3. Anderson KA, Burbach JA, Fenton LJ, Jaqua RA, Barlow JF. Idiopathic arterial calcification of infancy in newborn siblings with unusual light and electron microscopic manifestations. *Arch Pathol Lab Med* 1985; **109**(9)**:** 838-42.

4. Attia TH, Abd Alhamed MM, Selim MF, Haggag MS, Fathalla D. Idiopathic Arterial Calcification of Infancy: Case Report. *J Radiol Case Rep* 2015; **9**(11)**:** 32-40.

5. Azancot A, Diehl R, Dorgeret S, Sebag G, Baumann C, Vuillard E *et al.* Isolated pericardial effusion in the human fetus: a report of three cases. *Prenat Diagn* 2003; **23**(3)**:** 193-7.

6. Bacon JF. Arterial Calcification in Infancy. *JAMA* 1964; **188:** 933-5.

7. Barlow JF, Fraser B, Carter GA. Aortitis and large vessel arteritis in a newborn. *Hum Pathol* 1985; **16**(4)**:** 427-9.

8. Barson AJ, Campbell RH, Langley FA, Milner RD. Idiopathic arterial calcification of infancy without intimal proliferation. *Virchows Arch A Pathol Anat Histol* 1976; **372**(2)**:** 167-73.

9. Bellah RD, Zawodniak L, Librizzi RJ, Harris MC. Idiopathic arterial calcification of infancy: prenatal and postnatal effects of therapy in an infant. *J Pediatr* 1992; **121**(6)**:** 930-3.

10. Beukes CA. Idiopathic arterial calcification associated with infantile aortic coarctation. *Histopathology* 1985; **9**(9)**:** 1017.

11. Bird T. Idiopathic arterial calcification in infancy. *Arch Dis Child* 1974; **49**(2)**:** 82-9.

12. Bolster F, Ali Z, Southall P, Fowler D. Generalized arterial calcification of infancy--Findings at post-mortem computed tomography and autopsy. *Forensic Sci Int* 2015; **254:** e7-12.

13. Brachet C, Mansbach AL, Clerckx A, Deltenre P, Heinrichs C. Hearing loss is part of the clinical picture of ENPP1 loss of function mutation. *Horm Res Paediatr* 2014; **81**(1)**:** 63-6.

14. Byard RW. Idiopathic arterial calcification and unexpected infant death. *Pediatr Pathol Lab Med* 1996; **16**(6)**:** 985-94.

15. Cansu A, Ahmetoglu A, Mutlu M, Guven S, Osmanagaoglu MA. Idiopathic infantile arterial calcification: prenatal diagnosis and postnatal presentation. *Clin Exp Obstet Gynecol* 2010; **37**(1)**:** 73-5.

16. Carles D, Serville F, Dubecq JP, Alberti EM, Horovitz J, Weichhold W. Idiopathic arterial calcification in a stillborn complicated by pleural hemorrhage and hydrops fetalis. *Arch Pathol Lab Med* 1992; **116**(3)**:** 293-5.

17. Chen H, Fowler M, Yu CW. Generalized arterial calcification of infancy in twins. *Birth Defects Orig Artic Ser* 1982; **18**(3B)**:** 67-80.

18. Cheng KS, Chen MR, Ruf N, Lin SP, Rutsch F. Generalized arterial calcification of infancy: different clinical courses in two affected siblings. *Am J Med Genet A* 2005; **136**(2)**:** 210-3.

19. Chong CR, Hutchins GM. Idiopathic infantile arterial calcification: the spectrum of clinical presentations. *Pediatr Dev Pathol* 2008; **11**(5)**:** 405-15.

20. Ciana G, Trappan A, Bembi B, Benettoni A, Maso G, Zennaro F *et al.* Generalized arterial calcification of infancy: two siblings with prolonged survival. *Eur J Pediatr* 2006; **165**(4)**:** 258-63.

21. Ciana G, Colonna F, Forleo V, Brizzi F, Benettoni A, de Vonderweid U. Idiopathic arterial calcification of infancy: effectiveness of prostaglandin infusion for treatment of secondary hypertension refractory to conventional therapy: case report. *Pediatr Cardiol* 1997; **18**(1)**:** 67-71.

22. Corbacioglu Esmer A, Kalelioglu I, Omeroglu RE, Kayserili H, Gulluoglu M, Has R *et al.* Prenatal ultrasonographic diagnosis of generalized arterial calcification of infancy. *J Clin Ultrasound* 2015; **43**(1)**:** 50-4.

23. Crade M, Lewis DF, Nageotte MP. In utero appearance of idiopathic infantile arterial calcification: ultrasound study of a 28-week fetus. *Ultrasound Obstet Gynecol* 1991; **1**(4)**:** 284-5.

24. Dayapala A, Rathnayake IL, Pethiyagoda P. A case of idiopathic arterial calcification of infancy diagnosed at autopsy. *Ceylon Med J* 2016; **61**(3)**:** 137-138.

25. Dlamini N, Splitt M, Durkan A, Siddiqui A, Padayachee S, Hobbins S *et al.* Generalized arterial calcification of infancy: phenotypic spectrum among three siblings including one case without obvious arterial calcifications. *Am J Med Genet A* 2009; **149A**(3)**:** 456-60.

26. Edouard T, Chabot G, Miro J, Buhas DC, Nitschke Y, Lapierre C *et al.* Efficacy and safety of 2-year etidronate treatment in a child with generalized arterial calcification of infancy. *Eur J Pediatr* 2011; **170**(12)**:** 1585-90.

27. Eronen M, Pohjavuori M, Heikkila P. Fatal outcome of two siblings with idiopathic arterial calcification of infancy diagnosed in utero. *Pediatr Cardiol* 2001; **22**(2)**:** 167-9.

28. Farquhar J, Makhseed N, Sargent M, Taylor G, Osiovich H. Idiopathic infantile arterial calcification and persistent pulmonary hypertension. *Am J Perinatol* 2005; **22**(3)**:** 121-5.

29. Ferreira CR, Ziegler SG, Gupta A, Groden C, Hsu KS, Gahl WA. Treatment of hypophosphatemic rickets in generalized arterial calcification of infancy (GACI) without worsening of vascular calcification. *Am J Med Genet A* 2016; **170A**(5)**:** 1308-11.

30. Freychet C, Gay C, Lavocat MP, Teyssier G, Patural H, Bacchetta J *et al.* [GACI syndrome: a case report with a neonatal beginning]. *Arch Pediatr* 2014; **21**(6)**:** 632-6.

31. Gabilan JC, Habib R, Lajouanine P, Canet J, Riahi M. [Infantile calcifying arteriopathy]. *Arch Fr Pediatr* 1966; **23**(2)**:** 179-97.

32. Galletti S, Nitschke Y, Malavolti AM, Aquilano G, Faldella G, Corvaglia L *et al.* Generalized Arterial Calcification of Infancy: Fatal Clinical Course Associated with a Novel Mutation in ENPP1. *JIMD Rep* 2011; **1:** 23-7.

33. Giovannoni I, Callea F, Travaglini L, Amodeo A, Cogo P, Secinaro A *et al.* Heart transplant and 2-year follow up in a child with generalized arterial calcification of infancy. *Eur J Pediatr* 2014; **173**(12)**:** 1735-40.

34. Glatz AC, Pawel BR, Hsu DT, Weinberg P, Chrisant MR. Idiopathic infantile arterial calcification: two case reports, a review of the literature and a role for cardiac transplantation. *Pediatr Transplant* 2006; **10**(2)**:** 225-33.

35. Gleason MM, Weber HS, Cyran SE, Baylen BG, Myers JL. Idiopathic infantile arterial calcinosis: intermediate-term survival and cardiac sequelae. *Am Heart J* 1994; **127**(3)**:** 691-5.

36. Gower ND, Pinkerton JR. Idiopathic Arterial Calcification in Infancy. *Arch Dis Child* 1963; **38:** 408-11.

37. Green DW, Laughlin WR. Pathological case of the month. Idiopathic arterial calcification of infancy. *Arch Pediatr Adolesc Med* 1996; **150**(1)**:** 101-2.

38. Greenberg SB, Gibson J. New findings in idiopathic arterial calcification of infancy detected by MDCT. *AJR Am J Roentgenol* 2005; **185**(2)**:** 530-2.

39. Guimaraes S, Lopes JM, Oliveira JB, Santos A. Idiopathic infantile arterial calcification: a rare cause of sudden unexpected death in childhood. *Patholog Res Int* 2010; **2010:** 185314.

40. Haas M, Niehues SM. [Idiopathic infantile arterial calcinosis]. *Rofo* 2011; **183**(12)**:** 1162-4.

41. Hahnfeld S. [Arteriopathia calcificans infantum associated with osteogenesis imperfecta congenita (author's transl)]. *Zentralbl Allg Pathol* 1981; **125**(1)**:** 31-3.

42. Hamazaki M. Idiopathic arterial calcification in a 3-month-old child, associated with myocardial infarction. *Acta Pathol Jpn* 1980; **30**(2)**:** 301-8.

43. Hault K, Sebire NJ, Ho SY, Sheppard MN. The difficulty in diagnosing idiopathic arterial calcification of infancy, its variation in presentation, and the importance of autopsy. *Cardiol Young* 2008; **18**(6)**:** 624-7.

44. Heuser CC, Puchalski M, Kennedy A, Sangle N, Manuck T, Andres R. Radiographic and pathologic evaluation of idiopathic infantile arterial calcification. *Obstet Gynecol* 2010; **115**(2 Pt 2)**:** 465-8.

45. Hunt AC, Leys DG. Generalized arterial calcification of infancy. *Br Med J* 1957; **1**(5015)**:** 385-6.

46. Iglesias-Platas I, del Rio R, Rodriguez XF, Pedrini M, Rebollo-Polo M, Iriondo M. Two new cases of idiopathic arterial calcification in the newborn: watch out for lineal calcifications in plain radiographs. *J Pediatr* 2012; **161**(4)**:** 767-767 e1.

47. Inamura N, Nakajima T, Kayatani F, Kawata H, Takeuchi M. Idiopathic arterial calcification in infancy with twin-twin transfusion syndrome. *Pediatr Int* 2003; **45**(4)**:** 481-3.

48. Inwald DP, Yen Ho S, Shepherd MN, Daubeney PE. Idiopathic infantile arterial calcification presenting as fatal hypertensive cardiomyopathy. *Arch Dis Child* 2006; **91**(11)**:** 928.

49. Jones DE, Pritchard KI, Gioannini CA, Moore DT, Bradford WD. Hydrops fetalis associated with idiopathic arterial calcification. *Obstet Gynecol* 1972; **39**(3)**:** 435-40.

50. Juul S, Ledbetter D, Wight TN, Woodrum D. New insights into idiopathic infantile arterial calcinosis. Three patient reports. *Am J Dis Child* 1990; **144**(2)**:** 229-33.

51. Kalal IG, Seetha D, Panda A, Nitschke Y, Rutsch F. Molecular diagnosis of generalized arterial calcification of infancy (GACI). *J Cardiovasc Dis Res* 2012; **3**(2)**:** 150-4.

52. Karthikeyan G. Generalized arterial calcification of infancy. *J Pediatr* 2013; **162**(5)**:** 1074 e1.

53. Kirchner SG, Heller RM, Kasselberg AG. Idiopathic arterial calcification of infancy. *South Med J* 1979; **72**(10)**:** 1328-30.

54. Knieling L, Bulgaru-Iliescu D, Pandelea O, Stan CI, Popescu DC, Knieling A. IDIOPATHIC INFANTILE ARTERIAL CALCIFICATION - CASE REPORT. *Rom J Funct & Clin, Macro &Microscop Anat & Anthropol* 2014; **13**(3)**:** 311-314.

55. Kusaba A, Koja K, Kina M, Furuyama M. Idiopathic arterial calcification in 9-year-old boy: a successful reconstruction for ilio-femoral occlusion. *Jpn J Surg* 1985; **15**(1)**:** 68-74.

56. Kutty S, Cava JR, Frommelt MA. Idiopathic infantile arterial calcification: a case report of prenatal and postnatal echocardiographic diagnosis. *Echocardiography* 2009; **26**(7)**:** 862-4.

57. Le Boulanger G, Labreze C, Croue A, Schurgers LJ, Chassaing N, Wittkampf T *et al.* An unusual severe vascular case of pseudoxanthoma elasticum presenting as generalized arterial calcification of infancy. *Am J Med Genet A* 2010; **152A**(1)**:** 118-23.

58. Levine JC, Campbell J, Nadel A. Image in cardiovascular medicine. Prenatal diagnosis of idiopathic infantile arterial calcification. *Circulation* 2001; **103**(2)**:** 325-6.

59. Li Q, Schumacher W, Jablonski D, Siegel D, Uitto J. Cutaneous features of pseudoxanthoma elasticum in a patient with generalized arterial calcification of infancy due to a homozygous missense mutation in the ENPP1 gene. *Br J Dermatol* 2012; **166**(5)**:** 1107-11.

60. Li Q, Baker J, Kowalczyk J, Jiang Q, Uitto J, Schachner L. Paediatric pseudoxanthoma elasticum with cardiovascular involvement. *Br J Dermatol* 2013; **169**(5)**:** 1148-51.

61. Liu CT, Singer DB, Frates R. Idiopathic arterial calcification in infancy. Report of a case in a premature fetus. *Arch Pathol Lab Med* 1980; **104**(11)**:** 589-91.

62. Lussier-Lazaroff J, Fletcher BD. Idiopathic infantile arterial calcification: roentgen diagnosis of a rare cause of coronary artery occlusion. *Pediatr Radiol* 1973; **1**(4)**:** 224-8.

63. Maayan C, Peleg O, Eyal F, Mogle P, Rosenmann E, Bar Ziv J. Idiopathic infantile arterial calcification: a case report and review of the literature. *Eur J Pediatr* 1984; **142**(3)**:** 211-5.

64. Marrott PK, Newcombe KD, Becroft DM, Friedlander DH. Idiopathic infantile arterial calcification with survival to adult life. *Pediatr Cardiol* 1984; **5**(2)**:** 119-22.

65. Mehta S, Sterba RJ, Boyle GJ, Aziz PF. Unusual cause of myocardial ischemia in an infant. *Congenit Heart Dis* 2013; **8**(3)**:** E81-4.

66. Meradji M, de Villeneuve VH, Huber J, de Bruijn WC, Pearse RG. Idiopathic infantile arterial calcification in siblings: radiologic diagnosis and successful treatment. *J Pediatr* 1978; **92**(3)**:** 401-5.

67. Milner LS, Heitner R, Thomson PD, Levin SE, Rothberg AD, Beale P *et al.* Hypertension as the major problem of idiopathic arterial calcification of infancy. *J Pediatr* 1984; **105**(6)**:** 934-8.

68. Miyai K, Ariyasu D, Numakura C, Yoneda K, Nakazato H, Hasegawa Y. Hypophosphatemic rickets developed after treatment with etidronate disodium in a patient with generalized arterial calcification in infancy. *Bone Rep* 2015; **3:** 57-60.

69. Moran JJ, Becker SM. Idiopathic arterial calcification of infancy; report of 2 cases occurring in siblings, and review of the literature. *Am J Clin Pathol* 1959; **31**(6)**:** 517-29.

70. Moran JJ, Steiner GC. Idiopathic arterial calcification in a 5-year-old child. A case report. *Am J Clin Pathol* 1962; **37:** 521-6.

71. Morton R. Idiopathic arterial calcification in infancy. *Histopathology* 1978; **2**(6)**:** 423-32.

72. Muller KM, Tiwisina M, Blaschke R, Bassewitz DB. Structural analysis of pulmonary vascular changes in a case of infantile calcifying arteriopathy. *Beitr Pathol* 1976; **157**(1)**:** 84-92.

73. Nael A, Siaghani PJ, Chen D, Romansky SG, Shane L. Idiopathic infantile arterial calcification: a possible cause of refractory cardiopulmonary failure in infancy. *Case Rep Pathol* 2014; **2014:** 189850.

74. Nagar AM, Hanchate V, Tandon A, Thakkar H, Chaubal NG. Antenatal detection of idiopathic arterial calcification with hydrops fetalis. *J Ultrasound Med* 2003; **22**(6)**:** 653-9.

75. Nagaraj BR, Jain P, Thomas DA, Raghu M. Sonological appearance of idiopathic arterial calcification in fetus: A rare case. *Indian J Radiol Imaging* 2009; **19**(3)**:** 248-51.

76. Nasrallah FK, Baho H, Sallout A, Qurashi M. Prenatal diagnosis of idiopathic infantile arterial calcification with hydrops fetalis. *Ultrasound Obstet Gynecol* 2009; **34**(5)**:** 601-4.

77. Numakura C, Yamada M, Ariyasu D, Maesaka A, Kobayashi H, Nishimura G *et al.* Genetic and enzymatic analysis for two Japanese patients with idiopathic infantile arterial calcification. *J Bone Miner Metab* 2006; **24**(1)**:** 48-52.

78. Otero JE, Gottesman GS, McAlister WH, Mumm S, Madson KL, Kiffer-Moreira T *et al.* Severe skeletal toxicity from protracted etidronate therapy for generalized arterial calcification of infancy. *J Bone Miner Res* 2013; **28**(2)**:** 419-30.

79. Pala HG, Bilgili G, Artunc Ulkumen B, Alkan F, Coskun S. A case of antenatal diagnosis and postnatal characteristics of idiopathic infantile arterial calcification (IIAC and prenatal diagnosis). *J Obstet Gynaecol* 2016; **36**(5)**:** 665-7.

80. Pao DG, DeAngelis GA, Lovell MA, McIlhenny J, Hagspiel KD. Idiopathic arterial calcification of infancy: sonographic and magnetic resonance findings with pathologic correlation. *Pediatr Radiol* 1998; **28**(4)**:** 256-9.

81. Parker RJ, Smith EH, Stoneman ME. Generalised arterial calcification of infancy. *Clin Radiol* 1971; **22**(1)**:** 69-73.

82. Pashankar D, Moore L. Test and teach. Number eighty three: Part 1. Idiopathic arterial calcification of infancy. *Pathology* 1997; **29**(2)**:** 175, 217.

83. Patel M, Andronikou S, Solomon R, Sinclair P, McCulloch M. Idiopathic arterial calcification in childhood. *Pediatr Radiol* 2004; **34**(8)**:** 652-5.

84. Perreault G, Bonin A, Davignon A. [Idiopathic arterial calcification in infants]. *Union Med Can* 1966; **95**(7)**:** 808-12.

85. Rain B, Martin E, Gabilan JC, Retbi JM, Dehan M. [Infantile calcifying arteriopathy, review of the literature and report of one case (author's transl)]. *Arch Anat Cytol Pathol* 1976; **24**(6)**:** 419-22.

86. Ramjan KA, Roscioli T, Rutsch F, Sillence D, Munns CF. Generalized arterial calcification of infancy: treatment with bisphosphonates. *Nat Clin Pract Endocrinol Metab* 2009; **5**(3)**:** 167-72.

87. Raphael SS, Horne WI, Hyde TA. Arterial medial calcification of infancy in brothers. *Can Med Assoc J* 1970; **103**(3)**:** 290-3.

88. Reitter A, Fischer D, Buxmann H, Nitschke Y, Rutsch F, Mottok A *et al.* Fetal hydrops, hyperechogenic arteries and pathological doppler findings at 29 weeks: prenatal presentation of generalized arterial calcification of infancy - a novel mutation in ENPP1. *Fetal Diagn Ther* 2009; **25**(2)**:** 264-8.

89. Retbi JM, Casasoprana A, Gabilan JC, Dehan M, Rosentstein-Retbi J. Idiopathic arterial calcification in infancy. A case report. *Eur J Pediatr* 1978; **129**(1)**:** 55-60.

90. Robinowitz M, Virmani R, McAllister H. Idiopathic arterial calcification of infants with calcific deposits in myocardium: A study of twelve autopsy patients [abstr]. *Circulation* 1980; **62:** III-116.

91. Rodriguez Fernandez LM, Alvaro Iglesias E, Nieves Diez C, Gomez Mora MJ, Garcia De La Fuente J. [Idiopathic arterial calcification in children: post-mortem diagnostic of a premature newborn]. *An Esp Pediatr* 2000; **52**(1)**:** 62-4.

92. Rosenbaum DM, Blumhagen JD. Sonographic recognition of idiopathic arterial calcification of infancy. *AJR Am J Roentgenol* 1986; **146**(2)**:** 249-50.

93. Rube C, Nerlich A, Spath A, Muller-Hocker J. [Arteriopathia calcificans infantum in an infant with mucoviscidosis]. *Pathologe* 1989; **10**(1)**:** 53-6.

94. Rutsch F, Schauerte P, Kalhoff H, Petrarulo M, August C, Diekmann L. Low levels of urinary inorganic pyrophosphate indicating systemic pyrophosphate deficiency in a boy with idiopathic infantile arterial calcification. *Acta Paediatr* 2000; **89**(10)**:** 1265-9.

95. Rutschow H. [Arteriopathia calcificans infantum (author's transl)]. *Geburtshilfe Frauenheilkd* 1976; **36**(11)**:** 969-72.

96. Ryerson LM, Chiletti R, Zacharin M, Tibballs J. Two cases of idiopathic infantile arterial calcification. *J Paediatr Child Health* 2010; **46**(12)**:** 777-9.

97. Saetung P, Punyathunya R. Idiopathic arterial calcification of infancy: a case report. *J Med Assoc Thai* 1995; **78**(7)**:** 369-73.

98. Saigal G. Idiopathic arterial calcification in infancy, pathology, diagnosis and treatment modalities. *Indian J Pediatr* 2002; **69**(3)**:** 265-7.

99. Saigal G, Azouz EM. The spectrum of radiologic findings in idiopathic arterial calcification of infancy: pictorial essay. *Can Assoc Radiol J* 2004; **55**(2)**:** 102-7.

100. Samon LM, Ash KM, Murdison KA. Aorto-pulmonary calcification: an unusual manifestation of idiopathic calcification of infancy evident antenatally. *Obstet Gynecol* 1995; **85**(5 Pt 2)**:** 863-5.

101. Samyn MM, Bick D, Humphrey JA, Gandy KL. Successful congenital heart surgery for a toddler with idiopathic infantile arterial calcification. *Pediatr Cardiol* 2010; **31**(7)**:** 1096-9.

102. Sarmila N, Joshi P, Vani R. Idiopathic Infantile arterial calcification –A Very rare case. *Online J Health Allied Sci* 2010; **9**(1)**:** 11-12.

103. Sawyer T, Stacey M, Mulreany M, Thompson M, Nitschke Y, Rutsch F *et al.* Generalized arterial calcification of infancy associated with meconium peritonitis: a case report and review of the literature. *Am J Perinatol* 2009; **26**(10)**:** 711-6.

104. Sebire NJ, Ramsay A, Sheppard M. Idiopathic arterial calcification presenting with cardiac failure and sudden death in an 11-year-old girl. *Pediatr Dev Pathol* 2002; **5**(4)**:** 412-4.

105. Shaireen H, Howlett A, Amin H, Yusuf K, Kamaluddeen M, Lodha A. The mystery of persistent pulmonary hypertension: an idiopathic infantile arterial calcification. *BMC Pediatr* 2013; **13:** 107.

106. Sholler GF, Yu JS, Bale PM, Hawker RE, Celermajer JM, Kozlowski K. Generalized arterial calcification of infancy: three case reports, including spontaneous regression with long-term survival. *J Pediatr* 1984; **105**(2)**:** 257-60.

107. Sladden RA. Coronary arteriosclerosis and calcification in infancy. *J Clin Pathol* 1952; **5**(2)**:** 175-82.

108. Spear R, Mack LA, Benedetti TJ, Cole RE. Idiopathic infantile arterial calcification. In utero diagnosis. *J Ultrasound Med* 1990; **9**(8)**:** 473-6.

109. Stanley RJ, Edwards WD, Rommel DA, Smithson WA. Idiopathic arterial calcification of infancy with unusual clinical presentations in sisters. *Am J Cardiovasc Pathol* 1988; **2**(3)**:** 241-5.

110. Stolte M, Jurowich B. [Arteriopathia calcificans infantum]. *Basic Res Cardiol* 1975; **70**(3)**:** 307-25.

111. Stryker WA. Arterial calcification in infancy with special reference to the coronary arteries. *Am J Pathol* 1946; **22:** 1007-31.

112. Stuart G, Wren C, Bain H. Idiopathic infantile arterial calcification in two siblings: failure of treatment with diphosphonate. *Br Heart J* 1990; **64**(2)**:** 156-9.

113. Sundaram S, Kuruvilla S, Thirupuram S. Idiopathic arterial calcification of infancy - a case report. *Images Paediatr Cardiol* 2004; **6**(1)**:** 6-12.

114. Thiaville A, Smets A, Clercx A, Perlmutter N. Idiopathic infantile arterial calcification: a surviving patient with renal artery stenosis. *Pediatr Radiol* 1994; **24**(7)**:** 506-8.

115. Thomas P, Chandra M, Kahn E, McVicar M, Naidich J, LaCorte M. Idiopathic arterial calcification of infancy: a case with prolonged survival. *Pediatr Nephrol* 1990; **4**(3)**:** 233-5.

116. Traisman HS, Limperis NM, Traisman AS. Myocardial infarction due to calcification of the arteries in an infant. *AMA J Dis Child* 1956; **91**(1)**:** 34-7.

117. Tran KH, Boechat MI. Idiopathic infantile arterial calcification: imaging evaluation and the usefulness of MR angiography. *Pediatr Radiol* 2006; **36**(3)**:** 247-53.

118. Tschumper B, Dietrich RB, Pais MJ, Crade M. Pediatric case of the day. Idiopathic infantile arterial calcification (IIAC). *Radiographics* 1994; **14**(3)**:** 675-7.

119. Vade A, Eckner FA, Rosenthal IM. Computerized tomography in occlusive infantile arteriopathy. *Pediatr Cardiol* 1989; **10**(4)**:** 221-4.

120. van der Sluis IM, Boot AM, Vernooij M, Meradji M, Kroon AA. Idiopathic infantile arterial calcification: clinical presentation, therapy and long-term follow-up. *Eur J Pediatr* 2006; **165**(9)**:** 590-3.

121. Van Dyck M, Proesmans W, Van Hollebeke E, Marchal G, Moerman P. Idiopathic infantile arterial calcification with cardiac, renal and central nervous system involvement. *Eur J Pediatr* 1989; **148**(4)**:** 374-7.

122. van Oort AM, Sengers RC, Stadhouders AM, ter Haar BG. Idiopathic arterial calcification of infancy. *Helv Paediatr Acta* 1979; **34**(4)**:** 369-74.

123. Van Reempts PJ, Boven KJ, Spitaels SE, Roodhooft AM, Vercruyssen EL, Van Acker KJ. Idiopathic arterial calcification of infancy. *Calcif Tissue Int* 1991; **48**(1)**:** 1-6.

124. Vera J, Lucaya J, Garcia Conesa JA, Aso C, Balaguer A. Idiopathic infantile arterial calcification: unusual features. *Pediatr Radiol* 1990; **20**(8)**:** 585-7.

125. Votava-Smith JK, Pitukcheewanont P, Randolph LM, Chmait RH. Generalized Arterial Calcification in a Recipient Twin: Discordant Fetal Hemodynamics Result in Differing Phenotypes in Monozygotic Twins with an ABCC6 Mutation. *Fetal Diagn Ther* 2017; **41**(3)**:** 234-236.

126. Wax JR, Blackstone J, Pinette MG, Cartin A. Hepatic vascular calcification: an early second trimester sonographic feature of idiopathic infantile arterial calcinosis. *Am J Obstet Gynecol* 2001; **185**(5)**:** 1267-8.

127. Weens HS, Marin CA. Infantile arteriosclerosis. *Radiology* 1956; **67**(2)**:** 168-74.

128. Whitehall J, Smith M, Altamirano L. Idiopathic infantile arterial calcification: sonographic findings. *J Clin Ultrasound* 2003; **31**(9)**:** 497-501.

129. Witzleben CL. Idiopathic infantile arterial calcification--a misnomer? *Am J Cardiol* 1970; **26**(3)**:** 305-9.

130. Yapicioglu-Yildizdas H, Ozbarlas N, Erdem S, Yilmaz MB, Ozlu F, Buyukkurt S *et al*. Two newborn babies with generalized arterial calcification of infancy, two new mutations. *Turk J Pediatr* 2016; **58**(4)**:** 419-423.

131. Yi Y, Tong T, Liu T, Lin Q, Xiong Y, Xu J. Prenatal diagnosis of idiopathic infantile arterial calcification without fetal hydrops. *Echocardiography* 2017; **34**(2)**:** 311-314.

132. Zhang E, Owen R, Bruce G, Wiebe S. Idiopathic infantile arterial calcification in a 12-year-old girl presenting as chronic mesenteric ischemia: imaging findings and angioplasty results. *Pediatr Radiol* 2011; **41**(11)**:** 1476-80.
