# Supplementary figures and images for: ENPP1-Fc prevents neointima formation in generalized arterial calcification of infancy through the generation of AMP
Source: Exp Mol Med. 2018 Oct 29;50(10):139. doi: 10.1038/s12276-018-0163-5 (PMC6204430; doi:10.1038/s12276-018-0163-5)

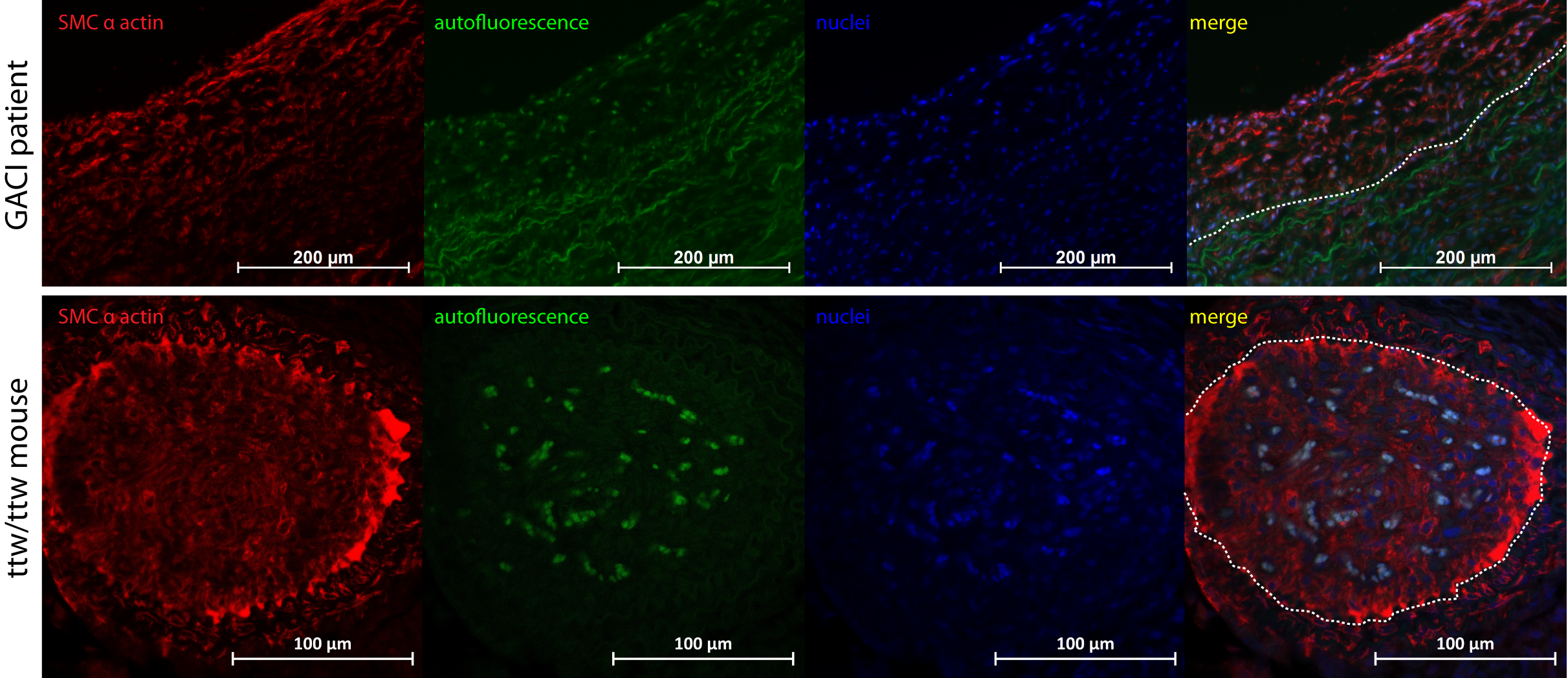

Supplement: Supplementary file 2 — Supplemental Figure 1 [file 12276_2018_163_MOESM2_ESM.tif]

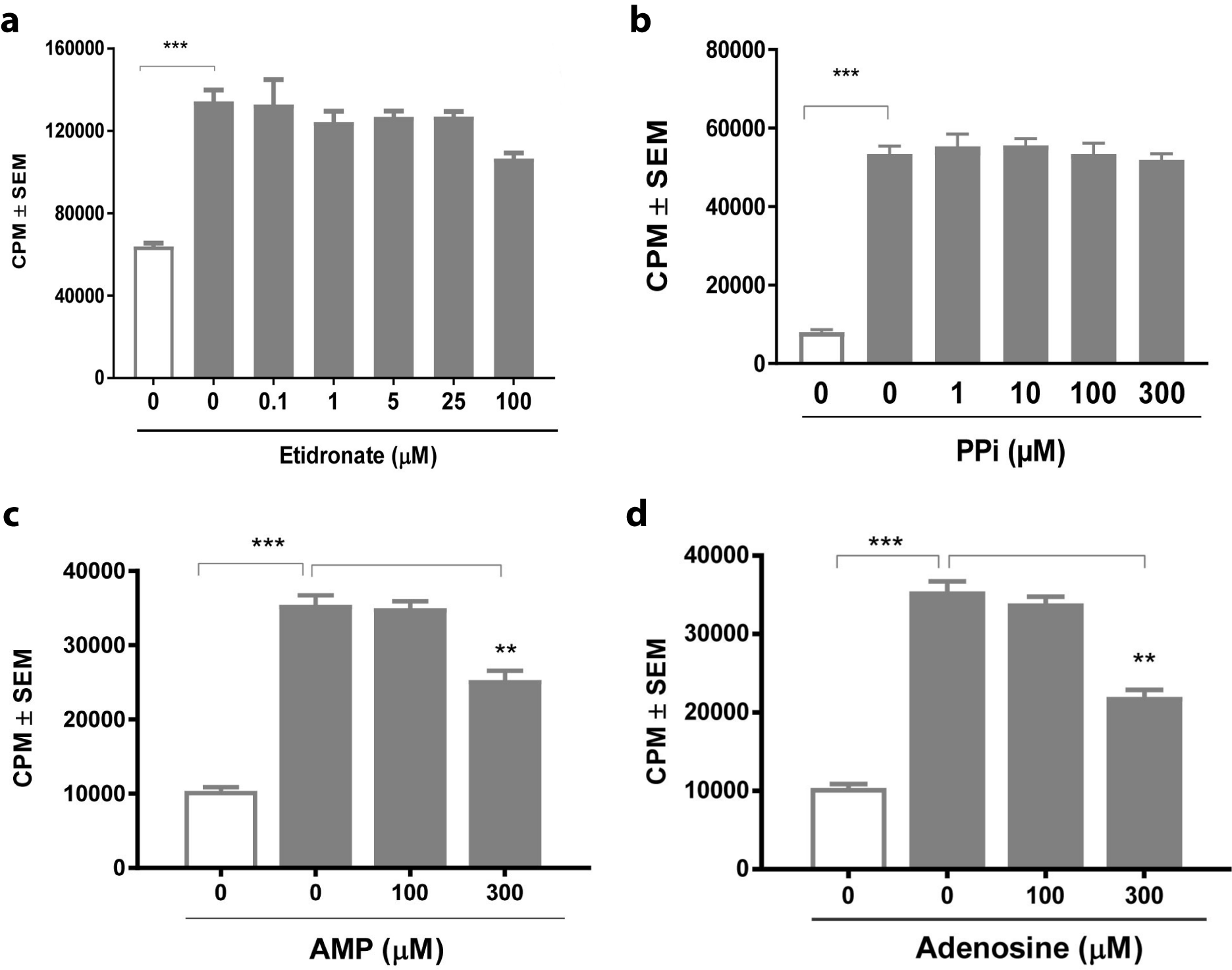

Supplement: Supplementary file 3 — Supplemental Figure 2 [file 12276_2018_163_MOESM3_ESM.tif]

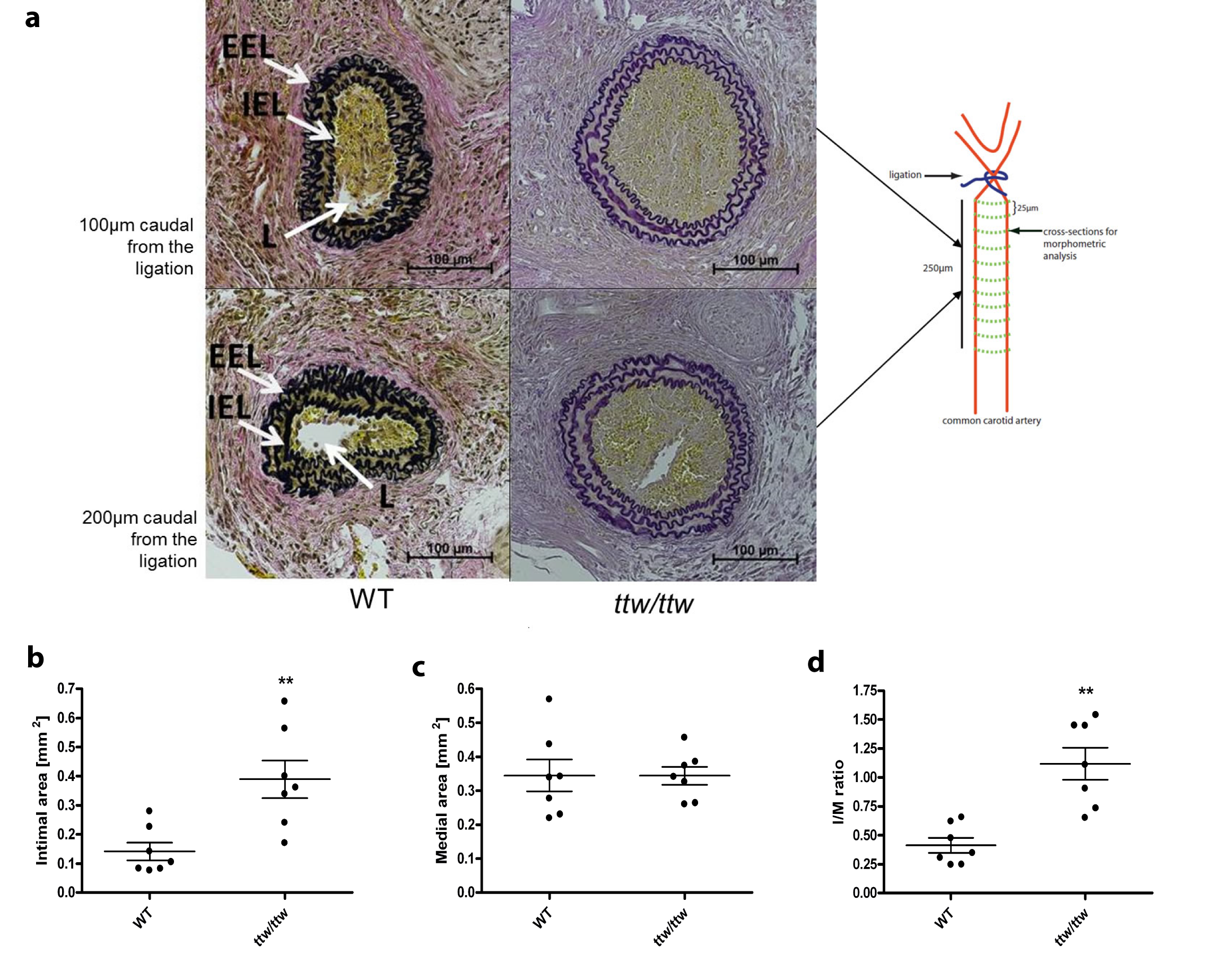

Supplement: Supplementary file 4 — Supplemental Figure 3 [file 12276_2018_163_MOESM4_ESM.tif]
